# Physical encounters impose a consistency–amount trade-off on bacterial group formation in marine environments

**DOI:** 10.1073/pnas.2521542123

**Published:** 2026-07-07

**Authors:** Thomas C. Day, Julia A. Schwartzman

**Affiliations:** ^a^ Department of Biological Sciences, University of Southern California, Los Angeles, CA 90089; ^b^ Department of Quantitative and Computational Biology, University of Southern California, Los Angeles, CA 90089

**Keywords:** group behavior, resource competition, ocean biophysics, bacteriophage, encounters

## Abstract

Physically associated bacterial groups can enhance resilience through emergent traits, including access to inaccessible substrates, increased stress tolerance, and reduced predation. These traits arise from physical and chemical interactions between cells and constitute multicellular behavior, yet we lack general principles predicting when this form of multicellularity provides ecological advantages. Here, we examine how the size of bacterial groups influence encounters with two particle types: resources and lytic bacteriophage. Using a size-dependent encounter theory, we identify regimes where encounter rates depend on group size. Experiments with the group-forming marine bacterium *Vibrio splendidus* 12B01 confirm that larger groups encounter particles more frequently. However, increasing group size reduces both the mean and variance of particle encounters per cell, revealing a trade-off between the consistency and amount of resource encountered. Stochastic simulations support this trade-off and predict conditions under which consistent encounters with resource particles benefit groups more than single cells, and where multicellular behaviors can alleviate resource limitation within groups. Simulations of encounters with phage-like particles predict that consistent encounters can stabilize the average time to infection for individual cells—the livable timescale—by disrupting viral replication and burst feedback. Overall, our results suggest that bacterial group formation can support resource acquisition despite predation. We predict that this advantage emerges in fluctuating fluid environments and is constrained by size- and concentration-dependent encounter physics. More broadly, these findings identify a simple physical mechanism through which multicellular groups may provide ecological advantages across diverse microbial systems.

## Significance

Physical associations among bacteria enable the emergence of group-level behaviors from cellular-scale interactions. Yet, predicting the ecological conditions in which physical associations advantage groups remains a challenge. Here we show how physical encounters in fluids can shape the costs and benefits of bacterial group living. Inspired by the microscale ecology of organic-matter degrading marine bacteria, we combine theory, experiments, and simulations to demonstrate how cells in larger groups experience more consistent, but less abundant, physical encounters with smaller particles and explore the ecological consequence of this trade-off for resource foraging and bacteriophage infection. We propose that the consistency–amount trade-off provides a physical framework to constrain the ecological contexts in which group formation is ecologically advantageous for bacteria.

The formation of physically associated groups—such as biofilms, bundles, and aggregates—can help bacteria overcome the ecological barriers that face single cells (1). Beyond conspicuous structures like fruiting bodies and filaments, group-level organization is a recurring motif in natural bacterial communities (2, 3). Traits like the metabolism of otherwise inaccessible substrates, increased resilience to abiotic stress, and resistance to predation emerge at the group level through behaviors such as division of labor and cell-type specialization, and are considered to be forms of early multicellularity (4). If we could describe the advantage of these traits in an ecological framework, we could better understand constraints on group formation, and perhaps how early multicellular groups evolve (5).

Marine environments concentrate many of the key ecological drivers of bacterial group formation. At the microscale, resources often occur in particulate form or are adsorbed onto particles, creating access challenges for individual cells (6). At the same time, the population dynamics of marine microbes are strongly influenced by predation from viruses and protists (7, 8). Far from static, coastal environments exhibit pronounced seasonality, exposing microbes to shifting environmental gradients over time (9). The combination of well-characterized environmental features and the diversity of microbial life makes coastal marine environments powerful natural model systems for studying the ecology and evolution of physically associated bacterial groups, and the multicellular behaviors they enable. However, the complexity of this environment also provides a challenge. Advancing the field will require frameworks that integrate the multiple drivers of multicellular group formation into a unified context, allowing us to evaluate the net ecological advantages of group formation.

Here, we ask how group size shapes encounters between heterotrophic bacterial degraders and two types of particles in marine systems: resources, and toxic particles like bacteriophage. Heterotrophic degraders form the foundation of microscopic detrital food webs in coastal ecosystems (10–12). These microbes break down substrates that span at least six orders of magnitude in size, from nanometer-scale organic acids to millimeter-scale marine snow (13). While interactions between bacteria and large organic particles are known as hotspots for microbial metabolism (14), a substantial fraction of organic matter exists in an intermediate size range (15). These particles are too large and chemically complex for direct uptake by single cells, yet too small to be effectively colonized (16). Moreover, this intermediate size class also includes toxic particles such as bacteriophage. We combine experiments and theory to investigate how multicellular group formation influences encounters with these intermediate-sized particles. Building on the framework of physical encounter kernels, previously used to model microbial interactions with resources and cells (17–21), we identify specific regimes where group formation is predicted to increase encounter rates. We further show that geometric constraints increase the consistency of encounters as a function of group size, and predict through modeling the ecological consequences of this trade-off in the context of resource competition and phage infection. Together, our work suggests that multicellular group formation may represent a prevalent but overlooked strategy for bacterial growth and survival in marine environments.

## Results

**Fig. 1.**
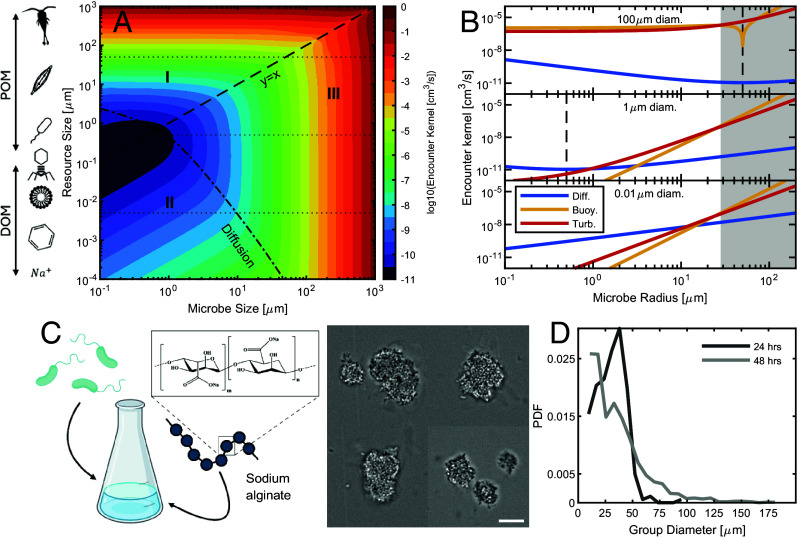
Size-dependent encounters between microbes and resources in coastal oceans predict an advantage for multicellular groups. (*A*) Predicted size-dependent physical encounter kernel, $\Gamma =\Gamma_{D}+\Gamma_{B}+\Gamma_{T}$, generated by separately varying the size of resources and the size of microbes. The dashed black line represents y=x, i.e. where resources and microbes are the same size; the dot-dashed line is the contour underneath which diffusion is the dominant contributor to the net kernel. These two contour lines divide the phase space into major sections: We label the 3 largest as Regimes I, II, and III. Horizontal dotted lines are cross-sections shown in (*B*). (*B*) 1D cross-sections of previously derived (e.g. ref. 23) encounter kernel components in (*A*) separated into: diffusion (blue), buoyancy (yellow), and turbulent flow (red). We show previously known theoretical encounter kernels for resources of 100 μm diameter (*Top*), 1μm (*Middle*), and 0.01 μm (*Bottom*). The gray region indicates the regime where the encounter kernel of turbulent flows is not theoretically resolved (26, 27). Vertical dashed lines indicate where microbe size is equal to the resource size. (*C*) When grown on alginate as a particulate carbon source (*Left*), *V. splendidus* strains form multicellular groups (*Right*). (Scale bar, 10 μm.) (*D*) Size distribution (probability density function, PDF) of *V. splendidus* groups from fluorescence microscopy after 24 h (one biological replicate, N=256 groups) and 48 h (the same biological replicate, N=1,683).

All marine microbes physically encounter particles in the water column. To build intuition for how cell or group size contributes to these encounters, we extended an existing encounter kernel framework for marine environments (22, 23) to consider how particles of different sizes are encountered by microbes ranging in diameter from submicrometer to hundreds of micrometers. This size range spans from picocyanobacteria to protists, and includes microbes that form aggregates and multicellular groups. The expected rate of geometric encounters between two species i and j is:

[1] $$\begin{array}{c} E_{\mathrm{ij}}=n_{i}n_{j}\Gamma , \end{array}$$

where $n_{i}$ and $n_{j}$ are the number concentrations of the species i and j, and Γ is called the “encounter kernel.” The kernel represents the accumulated effects of different biophysical processes that lead to encounters, including diffusion (D), buoyancy (B), and turbulence (T), and has previously been derived for each process individually (at least in limiting cases, for example, ref. 23). In general, the net encounter kernel is not known, and the coupling between these processes is still an active area of research (24, 25). We therefore consider the simple scenario where these biophysical processes are independent and uncoupled to each other. Then, we may build a linear superposition of these processes, $\Gamma =\Gamma_{D}+\Gamma_{B}+\Gamma_{T}$. Using the known encounter kernels for these strictly physical processes, we generated a size-dependent heatmap under the hypothesis that these processes are uncoupled (Fig. 1*A* and *SI Appendix*, section 1.1). Although encounter kernels for biological processes such as swimming can also be included, initially we omitted these to focus solely on the effects of size. The encounter kernels vary depending on physical factors like the intensity of turbulence and the excess mass density between the species i and j. We modeled an energy dissipation of $10^{-5}\;\text{W}{\text{kg}}^{-1}$, which is typical of sheltered coastal zones (28, 29), and considered the case where particles and microbes are the same density, with an excess density to seawater of $\Delta \rho =25\;{\text{kg m}}^{-3}$, a value typical of marine microbes and marine snow (*SI Appendix*, *Varying Density*) (30, 31).

This size-dependent view of encounters predicts three regimes (Fig. 1*A*). In the first regime, particles are larger than microbes, and the gradient of the encounter rate is much stronger along the size of the particle, suggesting that particle size is a more important parameter than microbe size. Regime I includes well-studied dynamics such as colonization of millimeter-scale marine snow (32–34). In the second regime, both interacting components are submicron scale, meaning that the net encounter kernel is dominated by diffusion (Fig. 1*B*). Microbes inhabiting regime II are known to often rely on chemotaxis to forage for ephemeral resource hotspots (18, 35–37). In the third regime, particles are strictly smaller than microbes, but both are large enough that diffusion is no longer the dominant contributor to the encounter kernel. The microbe’s size is predicted to contribute more to the net encounter kernel in this regime, as evidenced by the horizontal gradient in Fig. 1*A*. Well-characterized strategies of resource acquisition, such as the size-dependent feeding behaviors of ciliates, fall in this regime (38). Notably, regime III is also predicted to encompass a large fraction of interactions among bacterial groups and particles, including interactions spanning particulate and dissolved organic matter, and encounters with bacteriophage.

**Fig. 2.**
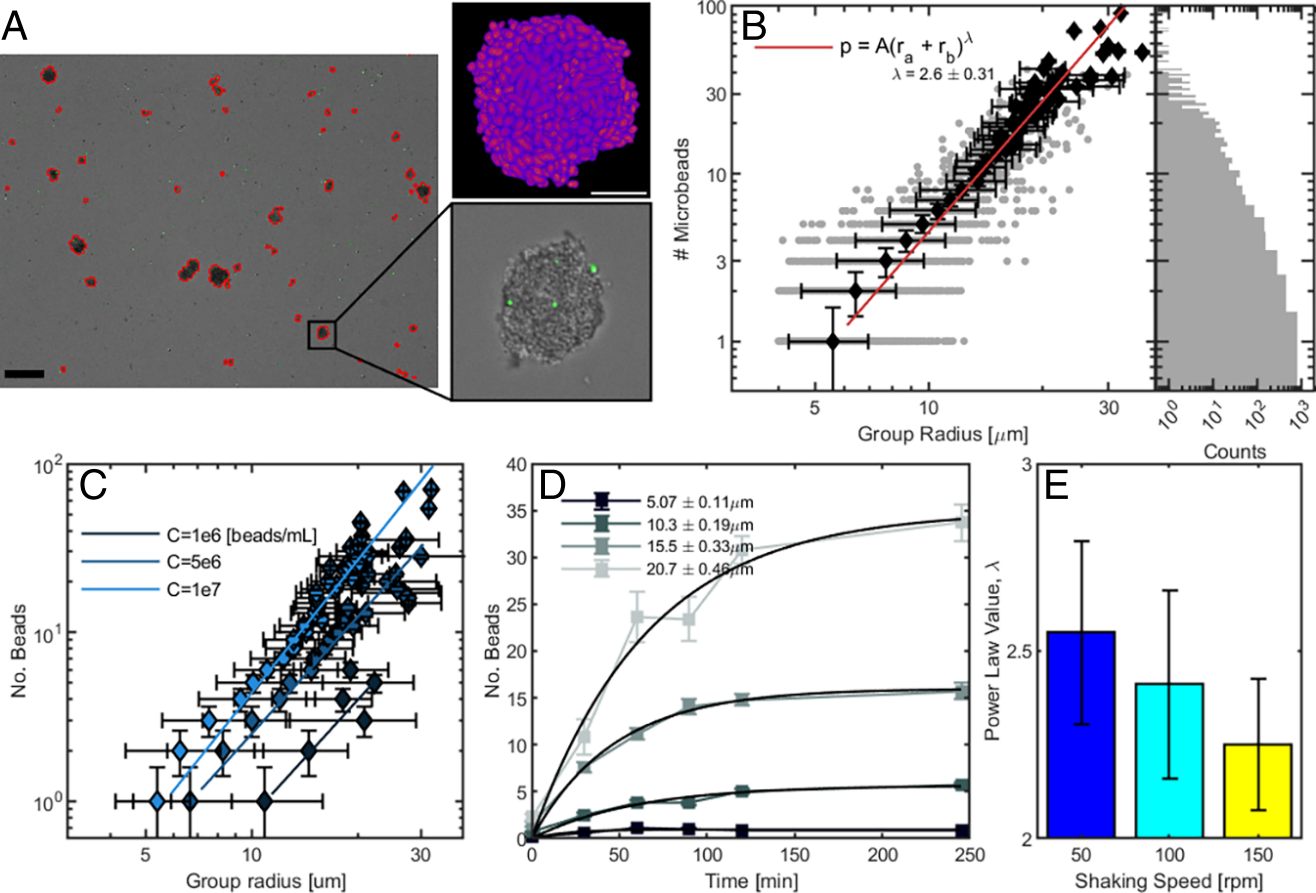
Larger multicellular groups encounter more particles. (*A*) Example microscope image, showing multicellular groups (DIC), overlaid with the green fluorescent channel showing microbeads (*Lower Inset*). Also overlaid in red are the boundaries from image segmentation. (Scale bar left is 100 μm.) *Upper Inset*: A 3D render of a confocal z-stack of one bacterial group, showing dense cell packing. (Scale bar is 5μm.) (*B*) Number of microbeads attached vs. group radius for one example treatment shaking at 100rpm for 90 min, with $n_{\text{beads}}=10^{7}\;\text{m}{\text{L}}^{-1}$. The number of groups observed in this single biological replicate was N=3,150. Gray points: individual counts of groups with the number of microbeads attached. Black diamonds: average size of groups with a given number of microbeads. Horizontal errorbars are the SD of group radii, vertical errorbars are an estimated error rate from hand-counting (*SI Appendix*). Red: power law regression to the mean with a 3σ confidence value. Side: Histogram of the number of groups counted with n microbeads. (*C*) Microbead attachment for different initial concentrations of fluorescent microbeads. Shown is one biological replicate, N={2,285,3,130,3,442} for $C=\{10^{6},5*10^{6},10^{7}\}$ beads/mL, respectively. (*D*) Average number of microbeads attached to groups of different size classes over time. Errorbars are the SE of the mean for groups in each size class/timepoint. Black lines are fits of a minimal attachment-detachment model (*SI Appendix*). Shown is one biological replicate sampled over time, for $10^{7}$ beads/mL. (*E*) The value of the power law exponent fit for 3 different shaking speeds tested of one biological replicate. Errorbars are the SE in the power law exponent from regression.

To test the prediction that particle encounters scale with microbial group size in regime III, we developed a simple experimental model (Fig. 1*C*) based on marine bacteria in shaken conditions that degrade the microscale polysaccharide alginate, a linear heteropolymer of uronic acids mannuronate and guluronate found in marine environments. Several strains of *Vibrio splendidus* form multicellular groups when cultured on polymeric alginate (39, 40). We selected a strain of *V. splendidus*, 12B01, that forms approximately spherical, tightly packed groups of cells (41). To measure encounters, we added monodisperse 1μm diameter fluorescent carboxyl-coated microbeads to shaken cultures of bacterial groups to simulate moderately turbulent conditions. The carboxyl coating of the microbeads was chosen to achieve efficient adsorption to cell groups (Fig. 2*A*). We also tested different surface chemistries by using amine-modified microbeads of the same size, and found no qualitative differences to our subsequent results (*SI Appendix*, Fig. S10). We found that the number of microbeads attached per group scaled as $P=A{\left(r_{a}+r_{b}\right)}^{\lambda }$ where $r_{a}$ is the group radius, $r_{b}$ is the microbead radius, and A and λ are fit parameters (Fig. 2*B*). Repeating this experiment with varying microbead starting concentrations revealed the prefactor A changed with microbead concentration, but the scaling relationship did not (Fig. 2*C*). Such a concentration-independent scaling relationship is consistent with the hypothesis that the primary factor determining microbead acquisition by bacterial groups is physical encounters, and also shows that in this system, we have avoided effects from surface area competition, which would flatten the relationship to scale quadratically. Together, these experiments demonstrate that in our experimental model, larger multicellular groups encountered more particles.

In addition to physical collisions, we considered the efficiency of microbead attachment and detachment, which are known important factors in encounter literature (34, 42), and contribute to the empirically measured kernel. We sampled encounters over time for a range of different bacterial group size classes (Fig. 2*D*). In all size classes, the rate at which microbeads accumulated on groups decreased over time, leading to a characteristic plateau of the number of microbeads attached per group. We derived a model of microbead attachment and detachment processes (*SI Appendix*, *Theory of Particle Attachment and Detachment*) that predicteds a functional form for this time-dependent relationship that depended on collision rate modified by attachment efficiency, $\alpha \Gamma n_{b}$, and detachment rate, β. We performed a nonlinear regression to the model, finding that the model fit this characteristic curve well (Fig. 2*D*). We found that while particle attachment rate was strongly size-dependent, as expected from our encounter theory, the detachment rate, β, was not significantly size-dependent. While further characterization of multicellular group properties is needed to determine the mechanisms through which encounter efficiency changes with group size, the data support the idea that size and concentration-dependent geometric encounters are the primary driver of differences in particle accumulation on bacterial groups in our model system.

While the value of the scaling exponent λ was empirically determined here, it is worth noting that in some cases, encounter theory predicts a known value for the scaling exponent. For the case where turbulence dominates the encounter kernel, there are simple scaling relationships derived when particles are either much larger or much smaller than the “Kolmogorov” length scale, $\eta_{K}$, which is the size of the smallest eddies in the turbulent fluid. When $r_{\mathrm{ab}}=\left(r_{a}+r_{b}\right)\ll \eta_{K}$, the predicted scaling relationship is λ=3 (22), and when $r_{\mathrm{ab}}\gg \eta_{K}$, λ=7/3 (43). We might therefore expect that, in the intermediate regime where $r_{\mathrm{ab}}∼\eta_{K}$, there would be a power law scaling exponent in the range 7/3<λ<3. This expectation is confirmed in previous numerical studies that explored the finite-inertia regime, which showed that as the particle size increased, the mean encounter rate normalized by the particle volume decreased (27). We found that the measured scaling exponent λ fit directly within the expected range from encounter theory. In real ocean surface systems, it is predicted that the Kolmogorov length will generally be larger than about 100 μm, and will generally be larger than 1 mm below about 10 m depth (29, 44). We cannot theoretically define the Kolmogorov length scale in our shaken culturing flasks, but we reasoned that if the Kolmogorov length scale contributed to the net encounter kernel scaling exponent, altering the shaking speed should change the net encounter kernel exponent. This is because the Kolmogorov length scale is inversely related to the intensity of turbulence in a fluid (43), which can be modified by changing the shaking speed. In our experiments, as shaking speed decreased, the empirically measured value of λ increased (Fig. 2*E*), indicating that turbulence plays a strong role in setting encounters in our system.

**Fig. 3.**
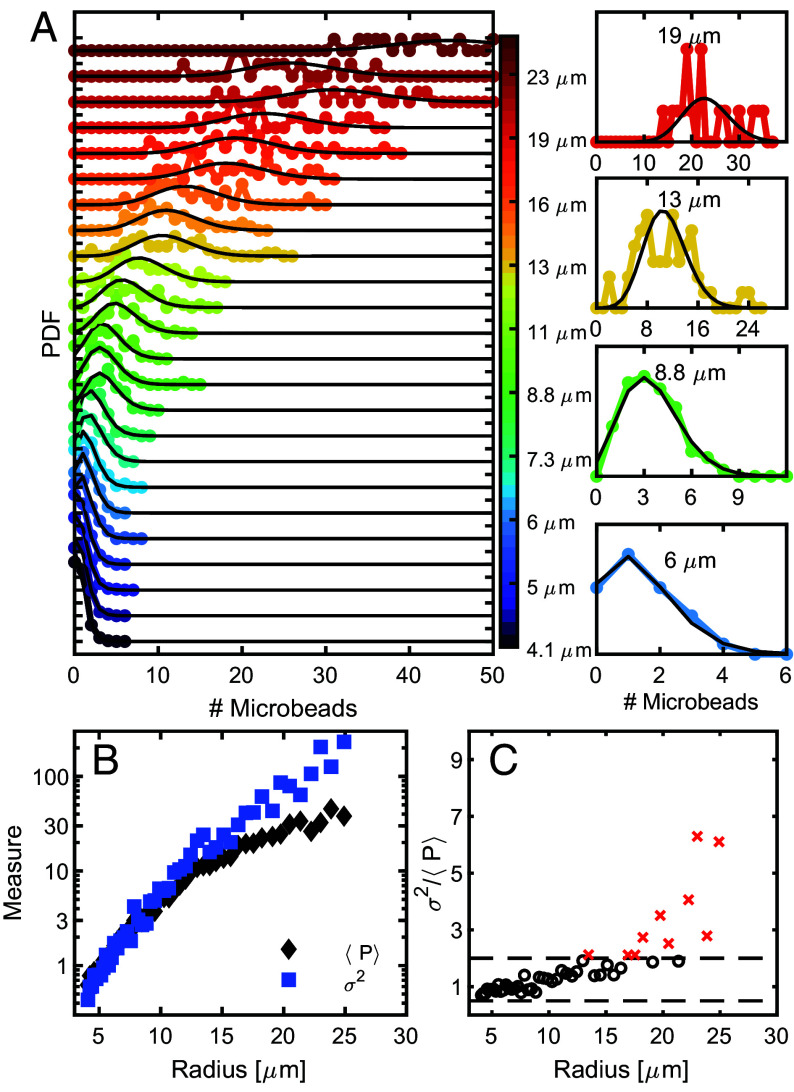
Encounters between particles and individual groups are Poisson-distributed. (*A*) Histograms of the number of microbeads attached to multicellular groups for the same biological replicate as shown in Fig. 2 at 120 min of incubation with microbeads. Each color histogram represents a different size class and is offset for visualization. Black lines represent Poisson predictions given the same mean as the size class. Thicker lines are size classes shown to the *Right*. PDF is the probability density function. *Right*, selected, representative histograms of the number of microbeads attached on multicellular groups for 4 different size classes. (*B*) Average (black diamonds, ⟨P⟩) and variance (blue squares, $\sigma^{2}$) in the number of microbeads attached vs. group size, plotted on the same y-scale. These values are extracted from the data in Fig. 3*A*. (*C*) Variance divided by mean for each size bin ($\sigma^{2}$/⟨P⟩). Horizontal dashed lines are min and max bounds of the chosen test statistic, at 0.5≤s≤2 where $s=\sigma^{2}/\left\langle P\right\rangle$. Points inside these bounds are displayed as black circles, outside as red x’s.

Having characterized the “mean-field” behavior of the net encounter rate, we next explored a defining feature of patchy, heterogeneous systems: fluctuations around the mean. If encounters occur independently and randomly, then the distribution of encounters would be Poisson (that is, the probability density function for k encounters is $f\left(k\right)=\gamma^{k}e^{-\gamma }/k!$ for each size class, where γ is the expected number of events). A Poisson prediction for the distribution of encounters in each size bin matched the data well for small groups (Fig. 3*A* and *SI Appendix*, Fig. S8), although there was a small deviation as groups become larger. Eventually, the mean and variance diverged when multicellular groups reached larger group sizes (Fig. 3*B*), starting around 15μm and larger (Fig. 3*C*). We found that a size-dependent deviation from Poisson predictions was consistent across different shaking speeds, flask sizes, and shaking modalities, although the exact size at which the mean and variance diverged varied in a condition-dependent manner (*SI Appendix*, Fig. S12). Contributing to this, we found a negative correlation between group size and group sphericity (*SI Appendix*, Fig. S13). Filtering aspherical groups from our dataset led to stronger agreement between the mean and variance in the number of microbeads attached across size bins. Together, these results suggest that microbeads on small multicellular groups are acquired in a manner that is close to Poisson, while larger multicellular groups may have distinct properties, such as aspherical shapes, that prompt larger deviations from the Poisson prediction.

**Fig. 4.**
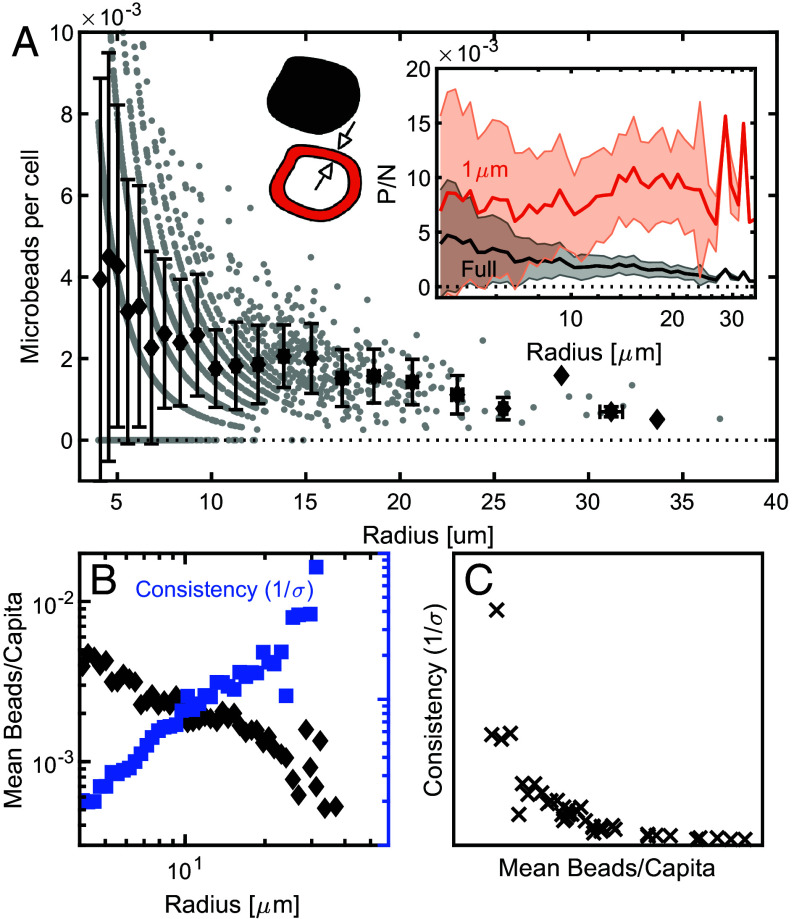
Per capita particles encountered suggest a size-dependent trade-off in the per capita rate and consistency of encounters. (*A*) Number of microbeads attached to a multicellular group per number of cells in the group. Gray points: individual instances, black diamonds: averages for different multicellular group size classes. Every fifth radius bin is shown for clarity. The horizontal black line represents encountering 0 microbeads per capita. Data are replotted from Fig. 2*A*. *Inset*: Estimated number of microbeads per active cell is plotted vs. group size, where active cells are those in a spherical shell closest to the group periphery, with shell thickness 1μm. Black line: the reference relationship where all cells in the group have access to nutrients. (*B*) Mean and consistency (inverse of the SD) of microbeads attached per capita from (*A*). (*C*) Consistency plotted vs. the mean number of microbeads per capita. The dashed black line represents neutral selection, i.e. no directional advantage.

How might a positive relationship between size and encounter rate shape the ecological dynamics of group-forming bacteria? To understand this question, we examined the per capita distribution of microbeads on multicellular groups. We first estimated the number of cells in multicellular groups of different sizes by enumerating the number of cells in 10 multicellular groups using confocal microscopy (*Materials and Methods*). We found that the volume of multicellular groups scaled linearly with the number of cells in the multicellular groups, allowing us to consistently convert apparent size via transmission microscopy to number of cells (*SI Appendix*, Fig. S11). We then converted our data (Fig. 2*B*) to a per capita distribution of microbeads, and found that while the mean number of microbeads per cell decreased with group size, so did the variance in the number of microbeads per cell (Fig. 4*A*). Put another way, smaller groups had a higher mean rate of microbead encounters per capita, but there was also high variation in whether any one group encountered microbeads. Many small groups encountered no microbeads over the measured time interval. Therefore, increasing size resulted in fewer, but more consistent, microbead encounters per capita (Fig. 4*B*). Intriguingly, the relationship between consistency and per capita amount appears concave (Fig. 4*C*), suggesting a trade-off that maximizes either of the extremes—larger cellular groups have high consistency but low numbers of microbeads per cell, and smaller cellular groups have lower consistency of encountering microbeads, but when they do, they encounter more per cell. Concave trade-offs are well known to drive specialization and coexistence of strategies.

We next asked how the trade-off between consistency and amount might affect resource acquisition by groups. Of course, not all cells in a group are likely to have equal access to nutrients. While the exact nature of group metabolism and resource sharing can be varied and complex, here we consider a simplified situation, where cells on the periphery of the group have more access to nutrients. If only cells in the group that actively consume resources are the subset of cells at the periphery, then we reasoned that the effective per-cell resource allocation would increase. We considered the case where a spherical shell of a few different thicknesses defines the active subset of cells, and rescaled the per-capita resource distribution accordingly (Fig. 4*A*). We find that for thin shells, the mean rate of geometric encounters per capita can remain constant or even increase with group size, at the same time that the consistency increases. In these cases, we predict that groups may mitigate the trade-off between consistency and amount.

**Fig. 5.**
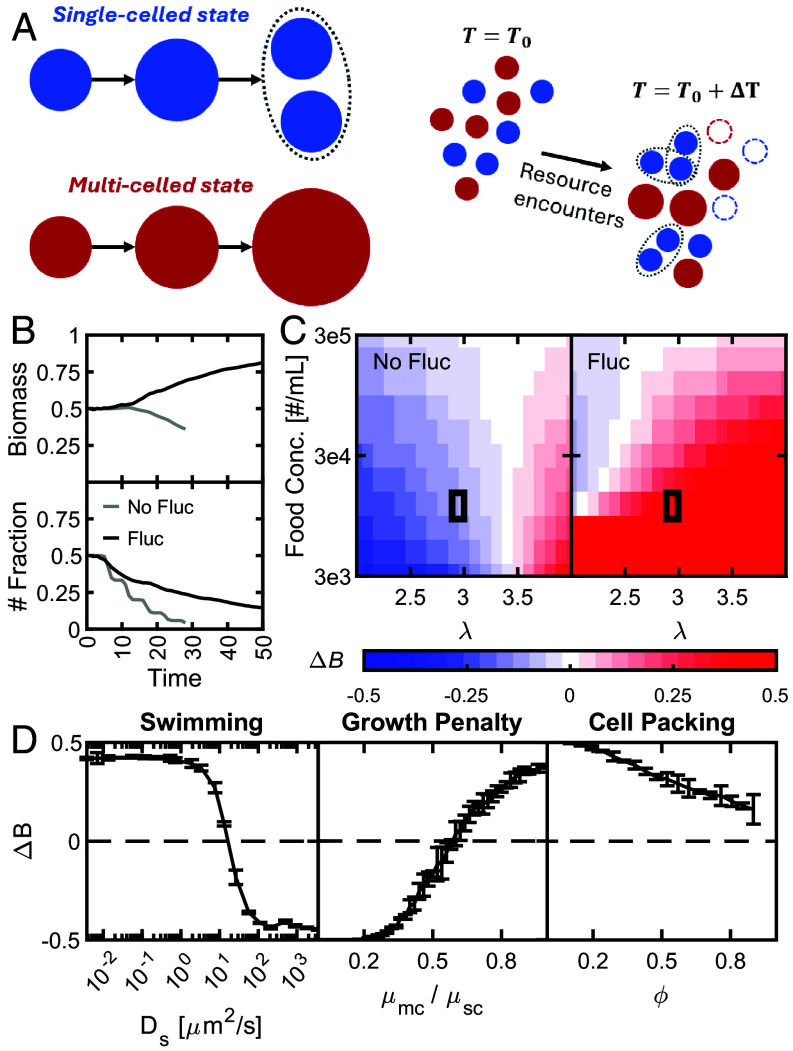
A model of encounter-driven resource competition predicts trade-offs between the mean-rate and the consistency of resource encounters define regimes where multicellular groups are the more competitive growth strategy. (*A*) Two different cell states in the model. The single-cell state divides when it reaches double its original size, the multicelled state does not divide, instead simply gaining biomass per individual. (*Right*) An illustration of how the simulation works in practice, illustrating division (dashed black outlines), growth (increase in size), and death (dashed outlines) as processes based on resource encounters. (*B*) Time tracks of the number fraction (*Bottom*) and biomass fraction (*Top*) of the multicellular state, for N=3 simulations for each type. Gray: simulation in only the mean field, i.e. not allowing for fluctuations in the number of encounters. Black: Tracking the multicellular state in a fluctuating, patchy environment. (*C*) A heatmap of the average change in biomass fraction (ΔB) of the multicellular state without (*Left*) and with (*Right*) fluctuations in the number of resources encountered, swept over the concentration of food and different values of λ, the net encounter kernel scaling exponent. Simulations from (*B*) are boxed. N=3 simulations for each parameter combination. (*D*) Change in biomass fraction (ΔB) for the multicellular state when including swimming for the single-celled state, growth penalties, and different cell packings. For each parameter set, N=3 simulations were averaged. Shown is 2σ=95% confidence bounds. Resource concentration was chosen such that the mean rate of encounters was $G_{0}=1.2*10^{-1}$ encounters per fastest doubling time for the single-celled state, and λ=2.7.

To understand the consequences of a trade-off between consistency and per capita resource encounters for the dynamics of resource competition, we developed a simple computational model to simulate the essential features of our experimental system: namely, fluctuations in resource encounters, a rate of encounters that is size-dependent, and the ability to grow as either individuals or multicellular groups (Fig. 5*A*). In this model, populations can exist as a mix of single cells (which we called state S1) and multicellular groups (state S2). In state S1, cells divide and separate after division. In S2, cells divide but do not separate, instead remaining in the group and increasing group size. The growth of both states is dependent on resource encounters via Monod kinetics. We simulated populations of single cells that were initially proportioned into equal halves occupying either the S1 or S2 state. We were interested in testing two predictions from our microbead experiments: a) that multicellular states can arise in a population that is competing for resources with single cells, and b) that the two states can coexist for significant periods, as indicated by the concave nature of the mean-rate/consistency trade-off.

Our primary goal was to compare mean-field and stochastic models to understand how fluctuations in encounters change the ability for single-celled and multicelled states to compete for resources. We swept two variables: the concentration of food particles c and the scaling exponent of resource acquisition, λ. A different value for the scaling exponent λ could be achieved by either a different environment (for example, a change in turbulence levels) or by a variable size-dependent attachment efficiency or detachment rate. When there were no fluctuations in resources (gray lines, Fig. 5*B*), the total biomass of S2 and the number of S2 groups both decreased due to resource outcompetition by the S1 state. We found that S1 outcompeted S2 in the mean field simulation over a wide range of resource concentrations and encounter scaling values (Fig. 5*C*). At very low resource concentrations (*SI Appendix*, Fig. S11), both S1 and S2 states died off. Regimes of coexistence only emerged after lambda was equal to or greater than 3, corresponding to conditions where there is less turbulence and/or more efficient resource encounters at larger sizes, and also scenarios where processes like sedimentation become more important.

Next, we introduced fluctuations in resource acquisition via Poisson sampling. In our simulations of fluctuating resource encounters (black lines in Fig. 5*B*), we found broad regimes in which S2 multicellular states increased in total biomass (Fig. 5*C*). Importantly, in all of these cases, while the biomass fraction increased, the number fraction decreased. This indicates that the S2 state did not outcompete S1 by driving it to extinction. Rather, the two states coexist—supporting our prediction from the concave trade-off between mean rate and consistency. These regimes emerged even as the total number fraction of S2 entities decreased, as in our model they have no mechanism for replicating. Notably, S1 could only outcompete S2 when resource concentrations were high and λ was low, corresponding to inefficient resource encounters or highly turbulent environments. Collectively, these simulations predict that the ability to form large groups, and experience consistent, but low per capita resources, may present an advantage for resource competition in many types of fluctuating environments.

To further understand how the properties of single-celled and multicelled states interact with stochastic encounters, we asked whether swimming in the S1 state could tip the competitive balance, and destroy S1 and S2 coexistence. We increased the encounter rate of S1 to emulate swimming by adding a nonchemotactic swimming term to the resource acquisition encounter kernel (*Materials and Methods*), which is relevant when resource particles are too large to be chemoattractants, but too small to be decomposed into source of small resource molecules that could meaningfully contribute to cellular metabolism. As examples of resources that lie in this size range, marine polysaccharides such as alginate, carrageenan, laminarin, and fucoidan are all typically between 10 and 10,000 kDa, thus being larger than typical chemoattractants (which are more like 100 to 1,000 Da). The strength of swimming is quantified by the effective diffusion coefficient, $D_{s}$, which we allowed to range from $3*10^{-3}$ to $3*10^{3}\;\mu {\text{m}}^{2}/\text{s}$. This is a common approach to modeling bacterial dispersion for various styles of motility, including run-reverse and run-reverse-flick motion often observed in motile marine bacteria (45, 46). In the case of bacteria exhibiting run-and-tumble motion, and reorienting at a rate of 1Hz, this effective diffusion range corresponds to swimming velocities from 0.1 to 100μm/s. If bacteria tumble more frequently, or swim via run-reverse-flick motion, they have a lower effective diffusion constant for the same swimming speed (46) (*SI Appendix*, Fig. S12). Recovering mean-field behavior (i.e., recovering a regime where S1 outcompetes S2) required a diffusive capacity of $17\;\mu {\text{m}}^{2}/\text{s}$, corresponding to swimming speeds of 7μm/s when reorientations occur once a second, and $v_{0}=50\;\mu \text{m}/\text{s}$ when reorientations occur at a rate of 10Hz (Fig. 5*D*).

We also tested two other scenarios that could weight a competition more toward the single-celled S1 strategy: density-dependent growth penalties and varied density of cell packing (Fig. 5*D*). We reasoned that both might affect growth and resource distribution in the multicellular S2 state, as local density can increase resource competition, and this effect could be exacerbated if cells are packed more densely. To add each effect to our model, we imposed a growth penalty, quantified as the ratio of the maximum growth rates between the two states, $\mu_{\mathrm{mc}}/\mu_{\mathrm{sc}}$, and included packing fraction, ϕ, which shifted the conversion for the number of cells in a group to the size of that group. A growth penalty changes per capita survival, while cell packing changes the per-capita resource encounter rate. We tested the same resource concentration and size-scaling exponent as the swimming case above. We found that the S2 state required a growth penalty of about 60% of the S1 state in order to destabilize regimes in which coexistence had been observed (Fig. 5*D*). However, even cell packing up to 90% of the total volume of the multicellular group could not destabilize coexistence. As a 90% packing density corresponds to a very dense packing, these simulations suggest that competition in our simulation is sensitive to growth penalties, but not cell packing.

Real marine environments contain a mix of resources and various kinds of toxic particles. Because our encounter-based framework is agnostic to particle type, toxic encounters—such as bacteriophage infections—may fundamentally constrain the emergence and persistence of multicellular strategies. We therefore explored whether toxic particles prevent multicellular states from arising or surviving in 3 ways. First, we extended our competition simulations to include toxic particles in addition to resources. Second, we estimated characteristic encounter timescales for groups of different sizes in environments with varying toxic particle concentrations. Third, we directly simulated encounters with nonreplicative toxic particles and replicative bacteriophage-like particles, where infection results in a burst of new particles, using a Gillespie scheme.

**Fig. 6.**
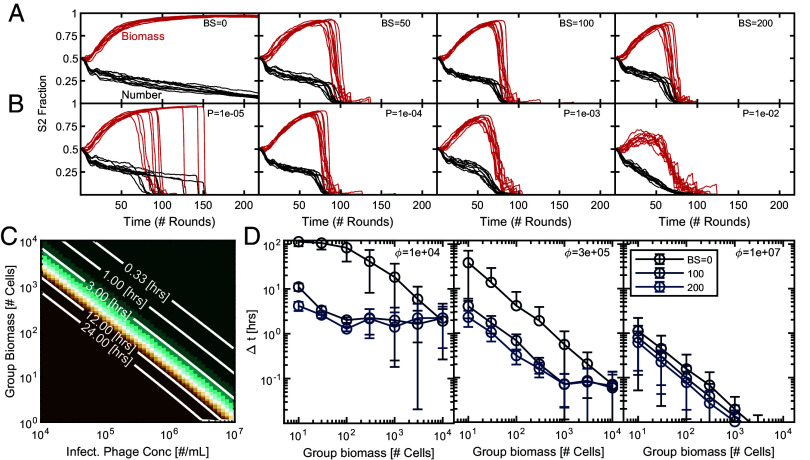
Toxic particles limit the livable timescale of multicellular strategies. (*A*) Biomass fraction (red) and number fraction (black) timetracks for state S2 in simulations sweeping different burst sizes (BS), representing the number of toxic particles released per capita upon infection. The initial concentration of toxic particles is held constant by a baseline rate of $10^{-4}$ particle encounters per cell doubling (P). (*B*) Timetracks for the same simulation held at BS = 100, varying baseline toxic particle encounters per cell doubling across 4 orders of magnitude. In both (*A* and *B*), baseline food encounters were held at 0.1 encounters per doubling. (*C*) Theoretical heatmap of the characteristic time before toxic particle encounter as a function of group size and toxic particle concentration, using the empirical encounter kernel from the microbeads experiments. (*D*) Average timescale before infection for individuals in a population, from Gillespie-style simulations of particle encounter, infection, lysis, and burst. Panels represent different toxic particle concentrations (ϕ) in mL-1.

In the extended competition simulations (Fig. 6 *A* and *B*), we found that multicellular states can form but are not viable in the long-time limit: Inevitable toxic encounters ultimately drive extinction. At sufficiently low toxic particle concentrations, however, multicellular groups can persist for extended but finite durations, which we term a “livable timescale,” denoted $\tau_{L}$, set by particle concentration and burst size.

We next estimated this livable timescale, as a function of toxic particle concentration, directly from our empirical encounter rates, yielding $\tau_{L}=1/\gamma ={\left(\Gamma n_{p}\right)}^{-1}$, where $n_{p}$ is the toxic particle concentration (Fig. 6*C*). We swept toxic particle concentrations between $10^{4}$ and $10^{7}$ particles per mL, typical values for the number concentration of phage in seawater (8). These estimates predict regimes of group size and particle concentration where the time to the first encounter between a toxic particle and a group is longest. This “livable timescale” was shortest for large groups at high toxic particle concentrations, ranging from minutes to hours, while smaller groups and low toxic particle concentrations allowed livable timescales on a timescale of days. These results provide a “worst case” estimate for persistence, and suggest that cellular groups large as thousands of cells may avoid encounter with bacteriophage for hours to days.

Finally, we implemented a Gillespie scheme simulation (*SI Appendix*, *Methods*) without cell growth to resolve individual encounter events between groups and toxic particles, and to account for dynamic changes to toxic particle concentration, so that we could study the effects of replicative phage-like behaviors of infection and burst size. We simulated a population of $10^{4}$ total cells, distributed into groups ranging from one group of $10^{4}$ cells to $10^{3}$ groups of 10 cells, suspended in 100μL of seawater, for a total concentration of $10^{5}$ bacteria per mL. This simulation revealed two key effects (Fig. 6*D*). First, increasing the initial concentration of toxic particle concentration sharply reduced livable timescales for all group sizes, as expected. Second, at lower initial concentrations of infectious particle (1× to 1,000× the concentration of groups in our simulations), burst size constrained livable timescales, especially for populations of many small groups. In these cases, replication of toxic particles from sparse early infections quickly amplified concentration of toxic particles, triggering a cascade of subsequent infections, and reducing the livable timescale by up to two orders of magnitude between burst sizes of 0 and 200. By contrast, a single large group experienced encounters near the mean rate; once infected, the population collapsed immediately. As a result, livable timescales for large groups were largely independent of burst size. Together, these simulations predict that populations that form groups increase the consistency of the timescale over which they encounter toxic particles, and that this timescale is less sensitive to the replication of toxic particles.

## Discussion

At the microscale, many ecological interactions are mediated by physical encounters. This is particularly true in aquatic environments, where encounters are shaped by size-dependent fluid dynamics. In this study, we highlight bacterial group formation as an important mechanism governing size-dependent encounters (Fig. 1). Combining experimental measurements and simulations, we predict that increasing bacterial group size drives more frequent total encounters, and more consistent encounters per capita. We used simulations to explore the consequences of this purely physical trade-off, and to predict that it can constrain the growth and survival of bacterial groups in encounter-driven environments. While here our focus is on understanding the dynamics of encounters from a physical standpoint, we highlight that the biological consequences of this physical constraint provide a useful framework for future study of the ecology of marine microbial populations. Indeed, this idea dovetails with the “foraging mandala” framework of Fernandez et. al., which ordinates bacterial resource foraging behaviors in terms of their search time and growth return (47). In this context, we propose that multicellular groups represent a potentially widespread, but understudied form of foraging behavior for marine bacteria.

When considering encounters between bacterial groups and resource particles, our work suggests that the consistency of encounters is an important parameter. In our simulations of resource competition, we predict a regime where forming groups—and thus increasing size—enhances a cell’s ability to encounter resources (Fig. 5). If resources are allocated evenly among cells, the geometric constraints imposed by group formation create a trade-off between encounter consistency and per capita resource availability (Fig. 4). However, such an even division is unlikely to occur in systems where cells at the group periphery have more access to resources than cells in the interior. As we demonstrate, resource allocation biased toward surface-associated cells can overcome this consistency–amount trade-off (Fig. 5). We further predict that this effect may be modified by changes in group architecture or inefficient resource recovery, as occurs for larger particles that require extracellular enzymatic degradation before transport (40, 48). Alternatively, cross-feeding interactions can allow some cells to proliferate on metabolic byproducts, retaining more resources in the population (49, 50). Collectively, these biological consequences of group formation fall into the framework of a multicellular group, in which division of labor allows groups to function differently from the sum of their individual cells. A limitation of the present work is that we do not specifically model differences in cellular-level physiology, such as differences in cell surface properties, which are known to affect the structure of bacterial groups (51, 52). However, by expanding the model that we present in the current study with more detailed experimental characterization, it will be possible in future work to understand how cells in multicellular groups physiologically respond to the trade-off between resource consistency and amount.

A complicating factor not interrogated exhaustively here is the shape of bacterial groups. Irregular shapes, even at fixed volume, anisotropically modify the effective radius of a group, leading to larger effective radii along some axes and smaller along others. In addition, group geometry can alter the surrounding hydrodynamic flows, potentially producing localized particle accumulation rather than uniform exposure (for example, ref. 24). This effect is likely to depend on particle size and could, in some cases, enhance attachment efficiency. Irregular morphologies may also arise through variation in cell packing density, persistent fluid flows (53), or local growth (54, 55). Taken together, these considerations suggest that more irregular shapes could increase the number of particles encountered on a per-capita basis. Changes in shape also affect total surface area, which in turn may influence the number of cells exposed to the environment, and thus participating in uptake or extracellular enzyme activity. This could plausibly impact overall growth rates. Future work should explore the connection between shape irregularity and encounter dynamics.

It is worth noting that swimming and chemotaxis are important and established mechanisms through which bacterial cells forage for resources (56). While we do not comprehensively compare chemotactic motility to group formation in this study, our simulations of resource competition predict that mechanisms to increase search area, such as motility, are an independent way to increase encounter consistency (Fig. 5*D*). In the case of motility, the energetics of foraging predict timescales over which cells can remain motile, that depend on the concentration of resources in the environment (47), and bacteria adopt distinct strategies based on their regulation of this motile timescale (57). Interestingly, many bacteria are capable of both chemotactic motility and cell–cell adhesion. We suggest that further studies comparing motility-based foraging and group-based foraging, and the transition between the two strategies, would expand our understanding of the spectrum of strategies through which the ecology of aquatic bacteria is shaped by physical encounters.

In this work, we consider a broader context of size-dependent physical encounters, including particles that can benefit or harm bacterial groups. In this context, the “livable timescale” emerges as a critical parameter for understanding the persistence of groups in the context of encounters with predators or toxins (Fig. 6). We specifically consider an example where bacterial groups and lytic bacteriophage are present in the same environment. At first glance, consistency appears inherently disadvantageous: The whole group will inevitably encounter a phage, which may infect it, propagate through it, and destroy it. How could a bacterial group ever survive in such an environment? Our simulations suggest that groups are at an inherent disadvantage: For a fixed toxic particle concentration, infection timescales will be shortest for populations that form larger but fewer groups. However, group formation also decreases the sensitivity of the infection timescale to the “burst” dynamics typical of a lytic bacteriophage infection. This is because bursts increase the concentration of phage, and therefore the encounter rate, so that as the burst size of a phage infection increases, the mean timescale for groups to encounter phage decreases. This dynamic, which emerges from a trade-off between encounter consistency and amount, shapes the livable timescale of a group—or the window of time that a group has to replicate or build phage defenses. While in this study we consider only a very idealized model of phage infection dynamics—an infection with perfect efficiency that lyses all cells encountered—bacteria encode a plethora of cellular-scale mechanisms to defend against phage, including at the group level, and the dynamics of phage infection themselves often do not directly result in lysis (58). Moreover, mechanisms of phage defense can include slower growth, suggesting that resource limitation in groups may impact infection dynamics. While in marine environments, bacteriophage outnumber bacterial cells ten to one (8), it is important to note that our framework can also encompass encounters with other types of nonresource particle, including bacterial predators (59), kin, and competing cells (50, 60, 61). Together, we suggest that expanding the encounter-driven framework to encompass resources, cells, and viruses could reveal key feedbacks that structure the dynamics of bacterial populations and that are relevant in real-world environments.

## Materials and Methods

### Data and Code.

All scripts used for image analysis, simulation scripts, and figure plotting scripts can be found here: (https://github.com/thomas-c-day/project-encounter.git). There is a permanent doi for the script repository via Zenodo: https://doi.org/10.5281/zenodo.20549471. All microscope images and simulation result files can be found archived on a Bioimage repository here: https://doi.org/10.6019/S-BIAD3600.

### Bacterial Cultures and Growth Conditions.

*V. splendidus* strain 12B01 (62) was routinely cultured in Marine Broth (Difco 2216), with 1.5% agar for solid medium. Cultivation was carried out at 25 ^°^C. Cultures of 12B01 grown as multicellular groups were established as described previously (41). Briefly, cells were grown as a preculture in 10mM glucose MBL minimal medium to an optical density (OD) measured at 600 nm of 0.2 to 0.5. OD measurements were taken using a Genesys 20 Spectrophotometer (Thermo Scientific). To prepare inoculum for cultures, cells were pelleted at 6000 rcf for 1 min in a tabletop microcentrifuge (Eppendorf), and resuspended in carbon-free MBL minimal medium at a final OD of 1.0. Experimental cultures were initiated by inoculating 12B01 at an initial density of $10^{-5}$ OD into 1.5mL of MBL minimal medium containing 0.07% w/v low viscosity alginate (Sigma A1112) in a 24-well-plate culture vessel (VWR 10861-558). Cultures were grown with shaking at 125 rpm on a Thermo Scientific MaxQ 2000 orbital shaker, unless otherwise specified.

### Microbead Incubation Experiments.

Bacterial groups were grown in liquid cultures overnight, such that cultures contained a range of bacterial group sizes. One micron diameter carboxyl-coated fluorescent microbeads (Fluospheres, Invitrogen, Lot # 2530763 for carboxyl beads, Fluospheres, Invitrogen, Lot # 3193678 for amine beads) were sonicated for 15 min at 40 KHz (Sonicator: Branson 5800) to minimize microbead–microbead adhesions. For experiments with amine-coated beads, the amine beads were sonicated in MilliQ water first, and then diluted and suspended in the minimal culturing media. We then mixed bacterial groups from culture and microbead suspensions at various relative concentrations. For the data shown in the main text, we used microbead concentrations of $c_{b}=\left\{10^{6},5*10^{6},10^{7}\right\}$ beads per milliliter, measured by diluting the suspensions from the manufacturer’s stated bead concentrations.

Incubation occurred in 1 well of a 24-well plate, filled with 1 mL of fluid for up to 245 min. The cultures of bacterial groups and microbeads were set on an orbital shaking platform at various speeds, as indicated. At each sampling point, we removed the culture from the shaking incubator and sampled 30 μm from the mixture onto a polymer-bottom well slide (Ibidi, Cat. No: 81826), then imaged. For experiments where we changed shaking speed, we separately varied the orbital shaking speed to be 50, 100, and 150 rpm for three separate cultures. For experiments where we changed the shaking type and container, we used three separate setups described here: First, we used the typical setup described above—1 mL culture with beads in a 24-well plate, shaken on the same orbital platform at 100 rpm. Second, we used a much larger volume of fluid—15 mL of bacterial culture contained in a 25 mL glass culture flask—set on the same orbital shaker at 100 rpm. Third, we used the typical volume and plate setup with a different shaker, a side-to-side shaker set at 107rpm (IKA-Werke HS501).

### Microbead Microscopy.

Images were taken with a Nikon Eclipse Ti2-E inverted microscope with 10× Nikon Plan Apo DIC air objective (NA 0.45) and an additional 1.5× zoom, for a net magnification of 15×, with a Orca Fusion C14440 digital camera and LED lightsource (Nikon D-LEDI). To ensure that all microbeads attached to the cell groups were visualized, we imaged multiple z-planes spaced at 5 μm intervals in both transmission DIC mode and in the green fluorescent channel (fluorescent excitation 488 nm). Each z-stack was then projected into a single image using the Nikon Elements software (AR 6.02.03) “Extended Depth of Focus” function, a method of obtaining a z-projection of maximum focus. The DIC (transmission) channel was segmented using a trained model of Ilastik (63) and custom-written MATLAB scripts (see *SI Appendix* for scripts) to generate the boundary of each aggregate larger than 4 μm in diameter. The fluorescent channel was segmented in MatLab (version R2024a) to find microbead centers, and the number of microbeads that resided within each aggregate boundary were enumerated.

In order to ensure that microbeads were not counted as a very small cell group with a microbead attached, we measured the total fluorescence per area of each group identified. If the value of the fluorescence per area exceeded $5*10^{3}$ units, we discarded the measurement.

### Measuring Number of Cells Per Group.

Cellular packing within individual groups was measured for 10 bacterial groups via confocal microscopy. Cell stain Syto-9 (Invitrogen) at 5 μM concentration was used to label individual cells of *V. splendidus* 12B01 grown on alginate for 36 h. We used a point-scanning confocal microscope (microscope body: Lecia DMI4000B, confocal unit: Leica TCS SPE) equipped with a solid-state 488 nm laser unit, and 2 photomultiplier tubes with an ACS APO 40×/1.15 NA CS series oil objective to image cells within groups with pixel resolutions between 0.135μm and 0.144μm per pixel. Z-stacks were taken with Δz = 0.40 μm, and the photomultiplier gain was set separately for each stack to maximize the dynamic range without saturation. After imaging, the xy and z resolutions were made equal using Fiji (64) release 2.15.0 and its plugin “Make isotropic.” Then, we used a custom Matlab script to segment the images, which included binarization, watershedding, and ellipsoid fitting.

### Cell State Competition Simulations.

We developed a simulation to model competition between two microbial strategies—single-celled and multicellular states—each drawing resources from a common pool. Individuals grow and decay based on the amount of food they acquire, like db/dt=(μ−δ)b, where b is the current total biomass of the individual, δ is a constant decay rate that penalizes starvation, and μ is a Monod-like growth rate that varies based on the per-capita food encountered. In the single-celled state, individuals divide once their biomass doubles; in the multicellular state, they do not divide and continue growing. Death occurs when biomass drops below a critical threshold. The simulation proceeds in rounds, with each individual’s biomass updated based on net growth, division, or death.

Food encounters were computed using empirically motivated encounter kernels incorporating diffusion, turbulence, and, in some cases, swimming. Baseline particle concentrations were varied by changing the baseline rate of encounters with groups of a single-cell size. Swimming was modeled as an effectively diffusive process, with a variable diffusion coefficient that depends on swimming speed and reorientation frequency. We simulated both deterministic and stochastic (Poisson-distributed) food availability, and explored a range of swimming strengths. To assess trade-offs, we varied the maximum growth rate of the multicellular state to introduce penalties relative to single-celled growth. Full simulation details, including equations and parameter sweeps, are provided in *SI Appendix*, *Methods*.

### Gillespie Simulation.

We explicitly resolved encounters between multicellular bacterial groups and phage via a Gillespie scheme neglecting bacterial growth. Populations were initially homogeneous, with each individual modeled as a spherical aggregate containing N cells. Group biomass is related to volume through the packing fraction $\phi =Nv_{c}/V$, where $v_{c}$ is the single-cell volume and V the aggregate volume. We set ϕ=0.4. Encounter rates are computed using the empirically measured encounter kernel from our microbead experiments and phage radius of 0.1 μm. For a population of M individuals, each individual i is assigned an encounter rate $r_{i}$, yielding a total rate $R=\sum_{i=1}^{M}r_{i}$. The time to the next encounter Δt is drawn from an exponential distribution with mean 1/R, and simulation time is updated as $t_{k}=t_{k-1}+\Delta t_{k}$. The individual experiencing the encounter is selected by sampling a uniform random variable over the population. Upon encounter, the selected individual is killed with probability q=1, and a phage burst of size $P=BS\times N_{i}$ is released. The phage concentration is updated as $\phi \left(t+\Delta t\right)=[\phi \left(t\right)v+BSN_{i}]/v$, where v is the surrounding fluid volume. The simulations run until either all individuals have been infected, or 120 h time limit is reached.

## Supplementary Material

Appendix 01 (PDF)

## Data Availability

Microscopy images, analysis code, and code for simulations have been deposited in Bioimage Archive (https://doi.org/10.6019/S-BIAD3600) (65) GitHub (https://github.com/thomas-c-day/project-encounter.git) (66) and Zenodo (https://doi.org/10.5281/zenodo.20549471) (67).
