# Supplementary material for: Physical encounters impose a consistency–amount trade-off on bacterial group formation in marine environments
Source: Proc Natl Acad Sci U S A. 2026 Jul 7;123(28):e2521542123. doi: 10.1073/pnas.2521542123 (PMC13367819; doi:10.1073/pnas.2521542123)
Supplement: Supplementary file 1 — Appendix 01 (PDF) [file pnas.2521542123.sapp.pdf]

Supporting Information: Physical encounters impose a consistency-amount tradeoff on bacterial group formation in marine environments

Thomas C. Day[a] and Julia A. Schwartzman[a,b]

[a]Department of Biological Sciences, University of Southern California
[b]Department of Quantitative and Computational Biology, University of Southern California

June 12, 2026

1 Theory of encounter rates

The rate of encounters between two particle species i and j can be modeled via:

$$E_{ij} = \Gamma_{ij} n_i n_j \tag{1}$$

where n_i is the concentration of species i in particles per unit volume. The encounter kernel Γ_{ij} has units of volume per time, meaning if a particle of type i (such as a group of bacteria cells) can "see" more fluid per unit time, then it will encounter more particles of species j (for example, a particle of food). The encounter kernel is affected by a variety of different kinds of biophysical processes, including particle diffusion, advection of the particle embedded in a fluid, buoyancy differences, and active motility. In turn, each of these terms of the net encounter kernel is affected by the particle sizes. Many of the physical processes that lead to encounters are derived in limiting cases (see [1, 2]); we reproduce them here for clarity.

The diffusion encounter kernel is:

$$\Gamma_D = 4\pi(D_i + D_j)(r_i + r_j) \tag{2}$$

where D_i and D_j represent the thermal diffusion constants, and r_i and r_j are the radii of the interacting components.

The buoyancy kernel is:

$$\Gamma_B = \pi(r_i + r_j)^2 |u_i - u_j| \tag{3}$$

where u_i represents the vertical velocity of particle i, and depends on particle density and size. If the vertical velocity u is described by Stoke's law, then $u(r_i) = 2\Delta\rho g r_i^2/9\mu$, and the encounter kernel will scale as the radius of the particle to the fourth power.

The turbulence kernel is:

$$\Gamma_T = 1.3(r_i + r_j)^3 \sqrt{\frac{\epsilon}{\nu}} \tag{4}$$

when the objects are much smaller than the Kolmogorov length scale. Here, ϵ represents the energy dissipation rate, and ν represents the kinematic viscosity of the fluid. The Kolmogorov length scale is also related to these parameters like $\eta = (\epsilon/\nu)^{1/4}$. When much larger than the Kolomogorov length scale, the turbulence kernel is instead [3]:

$$\Gamma_T = 1.37\pi\epsilon^{1/3}(r_i + r_j).^{7/3} \tag{5}$$

Note that these two functional forms do not intersect at the Kolmogorov length scale.

For ballistic swimming (i.e. swimming in a straight line), the encounter kernel is:

$$\Gamma_S = \frac{4}{3}\pi(r_i + r_j)^2 u \tag{6}$$

where u represents the swimming velocity of a motile cell in relation to a stationary particle. By inspection, we can see that this kernel scales quadratically in the size of the particles, but only linearly in the swimming speed. In this sense, all else being equal, changing size is a more important variable than changing swimming speed on the rate of encounters. Of course, microbes do not generally swim in a straight line. Often, they behave in a "run and tumble" fashion, where runs of roughly ballistic swimming are punctuated by brief periods of reorientation. The resulting trajectory is classically modeled as a random walk, at least in the case where there is no chemotaxis. Random walks have the same characteristics of thermal

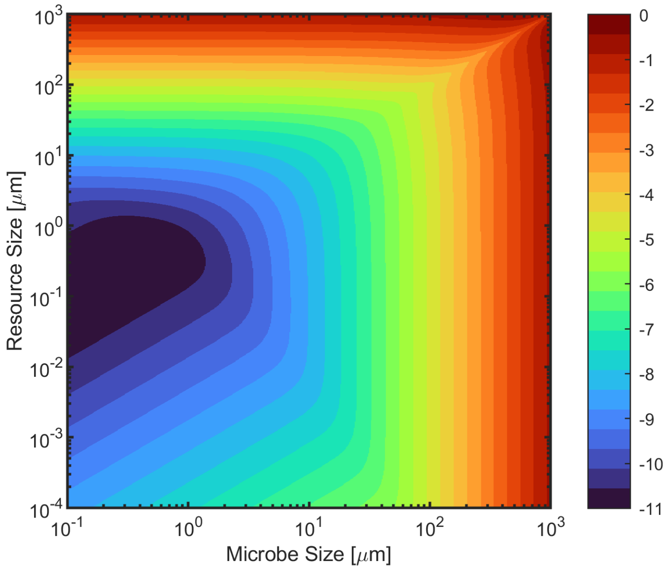

Figure S1: Adding kernels in quadrature shows the same qualitative behavior as linear addition.

diffusion, meaning that we can use the same form of the diffusion encounter kernel here, but with the thermal diffusion coefficients instead replaced by effective diffusion constants set by the mean squared displacement of bacterial swimming. The mean squared displacement is, in turn, set by run speed and run lengths. In later simulations of encounters via cell swimming, we will therefore approximate the encounter kernel like $\Gamma_S = 4\pi D_i(r_i + r_j)$ where D_i represents the effective diffusion constant of swimming, and is determined by the mean squared displacement of the random walk of bacterial run-and-tumble motion like: $\langle (x - \bar{x})^2 \rangle = 6D_i t$. When there is chemotaxis in swimming, the random walk formulation of swimming is not as appropriate.

1.1 Summation of encounter kernels

In general, it is not clear how to accumulate the different biophysical processes that lead to encounters into one net encounter kernel. If the processes are independent and uncoupled from one another, we may consider the linear addition of the kernels like so:

$$\Gamma_{ij} = \Gamma_D(r_i, r_j) + \Gamma_B(r_i, r_j) + \Gamma_T(r_i, r_j) + ... \tag{7}$$

However, some physical processes are known to be coupled to one another. For instance, coupling has been observed between turbulent flows and gravity in droplet collisions in clouds [4], between turbulent flows and sedimentation [5], and between particle shape and turbulent flows [6, 7]. In these cases, a simple linear sum is not sufficient to model the net encounter kernel. Other authors have suggested either summing the encounter kernels in quadrature [8], which doesn't seem to solve the coupling issue, or solving the equation of motion for each particle given the forces from each physical process [9], which may be computationally impossible, and at minimum neglects the power of the bulk encounter kernel approach.

We were interested to know if adding the kernels in quadrature would provide a qualitatively (or even quantitatively) different encounter heatmap. To test this, we modeled the encounter kernel for diffusion, turbulence, and buoyancy separately. Then, we element-wise divided each kernel by the matrix $R_{ij} = (r_i + r_j)^2$, so that $G = \Gamma/(r_i + r_j).^2$. This division gives an effective velocity for each kernel. Then, we performed the operation

$$G = \sqrt{G_D^2 + G_B^2 + G_T^2} \tag{8}$$

and re-multiplied element-wise by the matrix R_{ij}. This generated the heatmap in Figure S1, which is qualitatively the same as the one presented in the main text.

1.2 Varying the density of particles

Differences in buoyancy are an important driver of encounters in the ocean. The buoyancy encounter kernel is:

$$\Gamma_B = \pi(r_i + r_j)^2 |u_i - u_j| \tag{9}$$

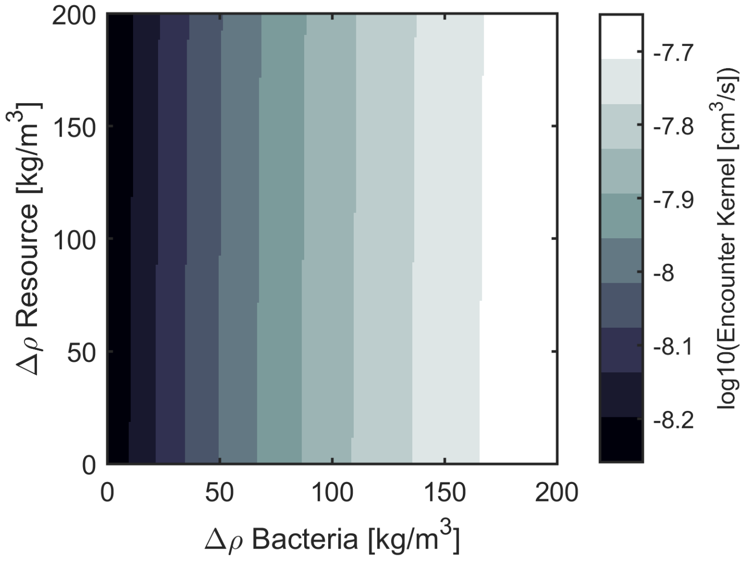

Figure S2: Net encounter kernel varies with changing particle densities. The net encounter kernel as a function of excess density (ρ) of 10 µm bacterial aggregates and 1 µm resource patches.

where r_i and r_j are the sizes of the interacting species, and u_i and u_j refer to their velocities in the water column, which can be either positive (for sinking particles) or negative (for rising particles). The velocity u_i is generally taken to be the terminal velocity of a (floating or sinking) object, and depends on particle density and shape. In the case of a small, spherical, sinking particle at low Reynold's number, the terminal velocity is $|u_i| = 2\Delta\rho g r.^2/9\nu$, where ν is the dynamic viscosity, g is the gravitational constant, r is the particle size, and $\Delta\rho$ is the difference in density between the particle and the water. Particles that are 10 µm in radius and 10 g/cm^3 in excess density of water approach their terminal velocity in less than ten-thousandths of a second, so the terminal velocity is quite a good approximation.

There is a broad range of particle densities in the ocean. Marine snow has been measured to have excess densities ranging from 10^{-5} to 10^{-1} g/mL [10], and similar values have been measured for living plankton [11]. For the main text Figure 1, we chose the middling value of $\Delta\rho = 0.025$ g/mL $= 25$ kg/m^3, and considered the case where microbial aggregates and resources shared the same excess density. We were interested in extending our analysis to consider situations where bacterial aggregates and resources do not share the same excess density. In Figure S2 we sweep a range of excess densities, from $0 \leq \Delta\rho \leq 200$ g/mL, separately for a bacterial aggregate 10 microns in radius and a resource patch 1 micron in radius, and calculate the expected net encounter kernel when $\epsilon = 10^{-5}$ W/kg. The encounter kernel varies by less than an order of magnitude from the extreme ends of the parameter sweep. We also found that the larger particle contributes more strongly to the gradient in the encounter kernel.

2 Theory of particle attachment and detachment

By observing the average number of beads per multicellular group after a certain time interval, we are observing the encounter rate modified by both an attachment efficiency and some detachment process. We write:

$$\frac{dp}{dt} = \alpha\Omega - \beta p \tag{10}$$

where p enumerates the average number of beads attached onto one multicellular group, α is the attachment efficiency of beads to multicellular aggregates, $\Omega = n_p\Gamma$ is the encounter rate of a multicellular group with any bead, n_p being the concentration of beads in the solution, and Γ representing the size-dependent geometric encounter kernel, and β is a detachment rate of beads from groups. We assume that α and β are independent of how many beads are attached to the group, and are independent of time. In general, both α and β can be size-dependent.

2.1 Particle attachment without any bead depletion effects

First, we consider the limit of high bead concentration, *i.e.* we do not consider dynamic changes to the concentration of beads that are not attached to any multicellular groups. We will write this as $n_p(t) = n_0$. Further, we assume that the multicellular group concentration changes negligibly in time.

The above equation is a linear differential equation of the form

$$\dot{p} + f(t)p = g(t) \tag{11}$$

which is exactly solvable. The solution is

$$p(t) = \frac{\alpha}{\beta}\Gamma n_0 + Ke^{-\beta t} \tag{12}$$

With the initial condition that $p(0) = 0$, we solve for the integration constant K, so that the final time-dependent result is

$$p(t) = \frac{\alpha}{\beta}\Gamma n_0(1 - e^{-\beta t}) \tag{13}$$

which can be confirmed as a solution to the differential equation above by substitution.

The variables α and Γ, representing the attachment efficiency and the encounter rate, appear together in this equation. Therefore, the attachment efficiency is not possible to measure from timetracks of the number of beads attached. We therefore combine the variables α and Γ in Equation 13 into one term (which we will call simply Γ), which represents the geometric encounter kernel modified by the attachment efficiency. There are therefore two free parameters in this equation: Γ and β.

2.2 Including the effect of microbead depletion during incubations

In the case where microbeads are constantly being depleted from the culture (for instance, because there is no influx of new microbeads), then the situation derived above changes to include information about the time-dependent microbead concentration. Here, we will show that the form of the solution to this situation is not dramatically different than that above, and approaches the same form as above in the limit of low concentration of multicellular groups.

We model the concentration of microbeads in the culture $n(t)$, which is a function of time as they get depleted. The concentration of microbeads attached to multicellular groups is $a = pc$, where p is the number of microbeads attached per multicellular group, and c is the concentration of multicellular groups. The total concentration of beads $N = n + a$ remains constant, *i.e.* microbeads either must exist in the culture medium or attached to multicellular groups. We can write this like:

$$0 = \frac{dN}{dt} = \frac{dn}{dt} + c\frac{dp}{dt} \tag{14}$$

The number of beads attached per multicellular group can be written, like above:

$$\frac{dp}{dt} = \alpha\Omega - \beta p \tag{15}$$

$$= \alpha\Gamma n - \beta p \tag{16}$$

Differentiate both sides to obtain

$$\ddot{p} = \alpha\Gamma\dot{n} - \beta\dot{p} \tag{17}$$

where we use dot notation for brevity. Substituting for $\dot{n}$ using Equation 14,

$$\ddot{p} = -(\alpha\Gamma c + \beta)\dot{p} \tag{18}$$

We solve this equation for $\dot{p}$, with the initial condition that $\dot{p}(0) = \alpha\Gamma N$.

$$\dot{p}(t) = \alpha\Gamma N e^{-(\alpha\Gamma c+\beta)t} \tag{19}$$

which we can integrate

$$p(t) = \int_0^t \dot{p}(t')dt' \tag{20}$$

$$= \frac{\alpha\Gamma N}{\alpha\Gamma c + \beta}(1 - e^{-(\alpha\Gamma c+\beta)t}) \tag{21}$$

In the limit where $\beta \gg \alpha\Gamma c$, then this approaches the solution of Equation 13.

We next sought to understand if it would be more valuable to include the depletion effect in our models. Using the parameters $\beta = 0.01\ \text{min}^{-1}$, and $\Gamma = 1 * 10^{-8}\text{mL}\,\text{min}^{-1}$, with 100% attachment efficiency, this would require a concentration of multicellular groups to be greater than 1 million groups per milliliter for the ratio $\alpha\Gamma c/\beta > 0.10$. If an OD 1 suspension of bacterial cells all clumped into groups with 1000 cells, then we expect the concentration of multicellular groups in suspension is likely to be at least one order of magnitude below this value. We therefore concluded that the non-depleting solution was adequate to model our dataset.

2.3 Competition for the surface area of the multicellular group

Let's consider that particles interact with the surface of a group. In high particle concentration limits, we might expect that competition for binding to the surface area plays an important role in limiting the attachment process.

Let there be a maximum occupancy per group that scales as its surface area, like $\sigma = \sigma_0 A_s$, where $A_s = 4\pi r^2$ is the surface area of the multicellular group, and σ_0 is the maximum occupancy density. Given a maximum packing fraction of particles on the surface ϕ, then the maximum surface density (number per unit area in square microns) is $\sigma_0 = \phi/a_0$, where $a_0 = \pi r_b^2$ is the maximum cross-sectional area of a particle. So the maximum total number of particles that can be attached to a surface is $\sigma = \phi A_s/a_0$. For microbeads with 1 micron diameter, we use the approximation that each bead sterically excludes a surrounding cylinder on a planar surface, and we will consider that particles in general can exclude space surrounding them in a similar fashion. Then, the maximum possible packing density of particles onto the surface of the group is close to $\phi \approx 0.9069$, which is the maximum packing fraction of disks in 2D. In reality, there are two corrections to make to this term: there may be fewer binding locations on the surface of the group (which will lower the maximum packing density), and the curvature of the group could allow for higher packing densities than a planar surface (which will increase the maximum packing density).

The rate of attachment of particles is then modified by the maximum occupancy fraction in a Langmuir-like way:

$$dp/dt = \alpha\Omega(1 - p/\sigma)$$

where p is the current number of beads attached. Note, here we have not included any bead detachment process, as we are just considering the attachment rate for now. The ratio p/σ, which is size-dependent, is an important parameter modifying the attachment rate of particles with groups. We plot this correction factor as a function of the group size and the number of beads attached, for 4 different packing fractions of beads on the group surface, ranging from $\phi = 0.01$ to $\phi = 0.9$, which approaches the maximum disk packing in 2D, in Figure S3.

We find that, at high ϕ, the ratio approaches a 10% correction factor only for very dense particle surface packings: for a 5 micron radius group, this equates to having 38 particles attached to the surface to have a 10% impact on the attachment rate. For a 10 micron radius group, 145 beads are required to have the same magnitude correction. For more moderate maximum packings $\phi < 0.9$, which could occur if there were specific binding sites that scaled with the group surface area, then the competition for these sites becomes stronger at lower numbers of attached beads: at 5 microns radius, this corresponds to 5 beads for a 10% effect, and at 10 microns radius, this corresponds to 17 beads, when $\phi = 0.1$. When there are a very low number of binding sites ($\phi = 0.01$), the surface competition becomes fierce: only one bead causes a 25% effect for a 5 micron radius group, and only 3 beads for a 10% effect at 10 microns radius.

In our system, we observe the following average numbers of beads for group radii of $R = \{5, 10, 20\}$ microns: $\{1, 3.6, 23.4\}$ after 60 minutes. For there to be a 10% effect from a surface site binding competition, the maximum packing fraction of beads on the surface would have to be between 0.02 and 0.03, if there are no bead detachment processes. As we have no evidence that there are particular spots on the surface of the multicellular group which are more amenable to binding the beads than any of the other area, and we also do not observe a significant flattening of the microbead-vs-size relationship, (Supplemental Figure S6, and we also do not observe flattening as we increase the particle concentration (Supplemental Figure S7), we rule out this surface area competition effect in our remaining model considerations.

2.4 Fitting the attachment-detachment model to microbead experiments

In our experimental data, we noted that the distribution of microbead attachment to larger multicellular groups was less well fit to our empirically measured encounter kernel. We sought to understand the main contributor to this size-dependent behavior of microbead attachments. To do this, we first considered the scenario where both Γ and β may be size-dependent. We used a nonlinear least squares regression to fit the model Equation 13 to experimental data via the following method. First, we binned timelapse data into distinct size bins (by group radius). We excluded size bins that had fewer than 10 instances. In each bin, the number of microbeads per multicellular group was averaged to obtain a series of timetracks, which we could fit with our model. We chose an initial parameter guess to be $\beta = 0.01\text{min}^{-1}$, and $\Gamma = 1 * 10^{-8}\text{mL min}^{-1}$ for each timetrack. We used MatLab's nonlinear fitting algorithm fitnlm. The respective fits for a few select size bins are summarized in Table S1, and the fits along with the time-tracks for various size bins are displayed in Figure S4a.

This fitting procedure found strong size-dependence for the fitting parameter Γ, representing the particle attachment rate, as expected from encounter theory. The Pearson's r-correlation coefficient was 0.97, and a Mann-Kendall trend test returned a very low p value of $1.6 * 10^{-8}$, indicating that a trend exists. The detachment rate, β, was of the order $\mathcal{O}(10^{-2})$, giving a characteristic timescale between 20 and 77 min for detachment rates for the values displayed in Table S1. It had a weaker, negative, size correlation, with a Pearson's r coefficient of -0.75, and a relatively higher Mann-Kendall p-value of $p = 2.1 * 10^{-5}$. However, as neither the Pearson's correlation nor the Mann-Kendall test take errors in the fit parameters into account, we sought to analyze the trend in β further.

We extended our analysis of the detachment rate, β, by examining the standard error in the fit value. For almost all size bins, $\beta > 0$ was found with high statistical significance ($p < 0.05$). The only exception was for groups of radii of

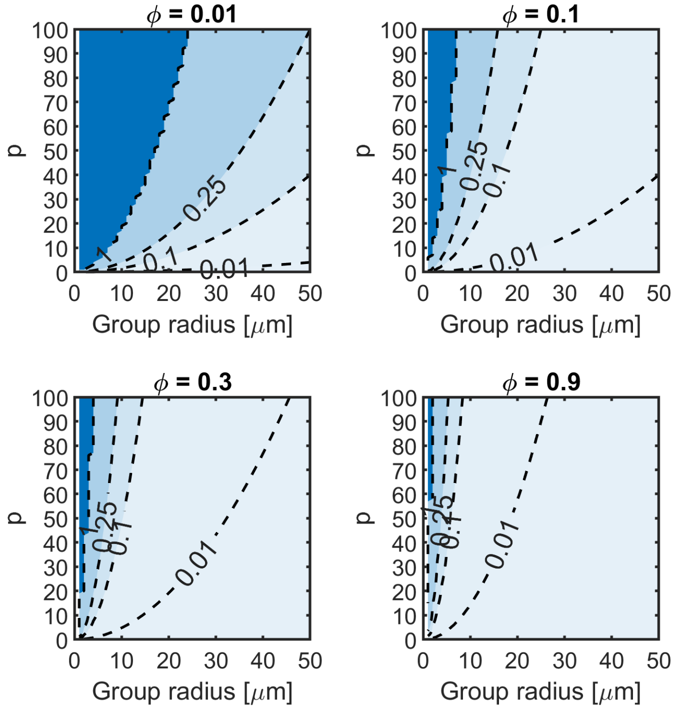

Figure S3: Corrections for competition over surface area.

R	β	Γ
5.0	0.050	$5.16 * 10^{-9}$
9.3	0.025	$1.01 * 10^{-8}$
15.7	0.020	$3.49 * 10^{-8}$
22.1	0.013	$5.29 * 10^{-8}$

Table S1: Fitting parameters for timetracks of different multicellular group sizes. R, size class measured as radius in µm; β, particle detachment rate measured as min^{-1}; Γ, measured as cm^3/min.

$R = 5.45 \pm 0.11$ µm, where $p = 0.15$. We can therefore conclude that a non-zero detachment rate is statistically likely. However, we noticed that the detachment rate for all groups larger than 10 microns in radius was quite flat. We averaged the detachment rate for $R > 10$ microns, finding that in this zone, $\bar{\beta} = 0.019\text{min}^{-1}$. We then used a two-sided t-test to compare the fit values of β for each size class with this average value. We found that this test failed to reject the null hypothesis at a $\alpha = 0.05$ significance level for 19/22 size classes: $R = \{5.1, 9.5, 15.6\}$ microns.

To understand how a constant β value might affect the timetrack fit, we performed fits to our model where we restricted the values of β and Γ. First, we held β to be a constant equal to the net average value $\bar{\beta} = 0.025\text{min}^{-1}$ from the fits to the timetracks above, so that the only free parameter was the collision and attachment kernel, Γ. We show the result of these fits in Figure S4b. When compared to fits where β was allowed to vary, we found that the sum of the root mean square of the residuals was consistently higher for all size classes (Figure S4d). However, on average, the sum of the rms residuals was about 130% of the value of the residuals from the full fit, indicating that the size-dependence of the detachment rate is only mildly important. By comparison, allowing β to vary, but fixing Γ to be the average of the fits from above, the sum of the root-mean-square of the residuals is on average about 270% higher.

In summary, we did not find enough statistical evidence to conclude that the particle detachment rate, β, is size-dependent. For the remaining regressions and analysis, we therefore proceeded using a constant β value.

2.5 Encounter rate regressions as a function of size

We next sought to measure the mean-field encounter rate as it varied with size. From Equation 13, given no size dependence for the parameter β, the only size-dependent parameter is the attachment-modified encounter kernel $\alpha\Gamma$. Because of this, and since encounter rate theory of the turbulence kernel both well above and well below the Kolmogorov microscale are power-law relationships, we used a power-law regression to fit the number of microbeads attached as a function of multicellular group size. In Figure 2, we show this regression on a log-log-scale. Additionally, here we show a linear-scale version of this regression

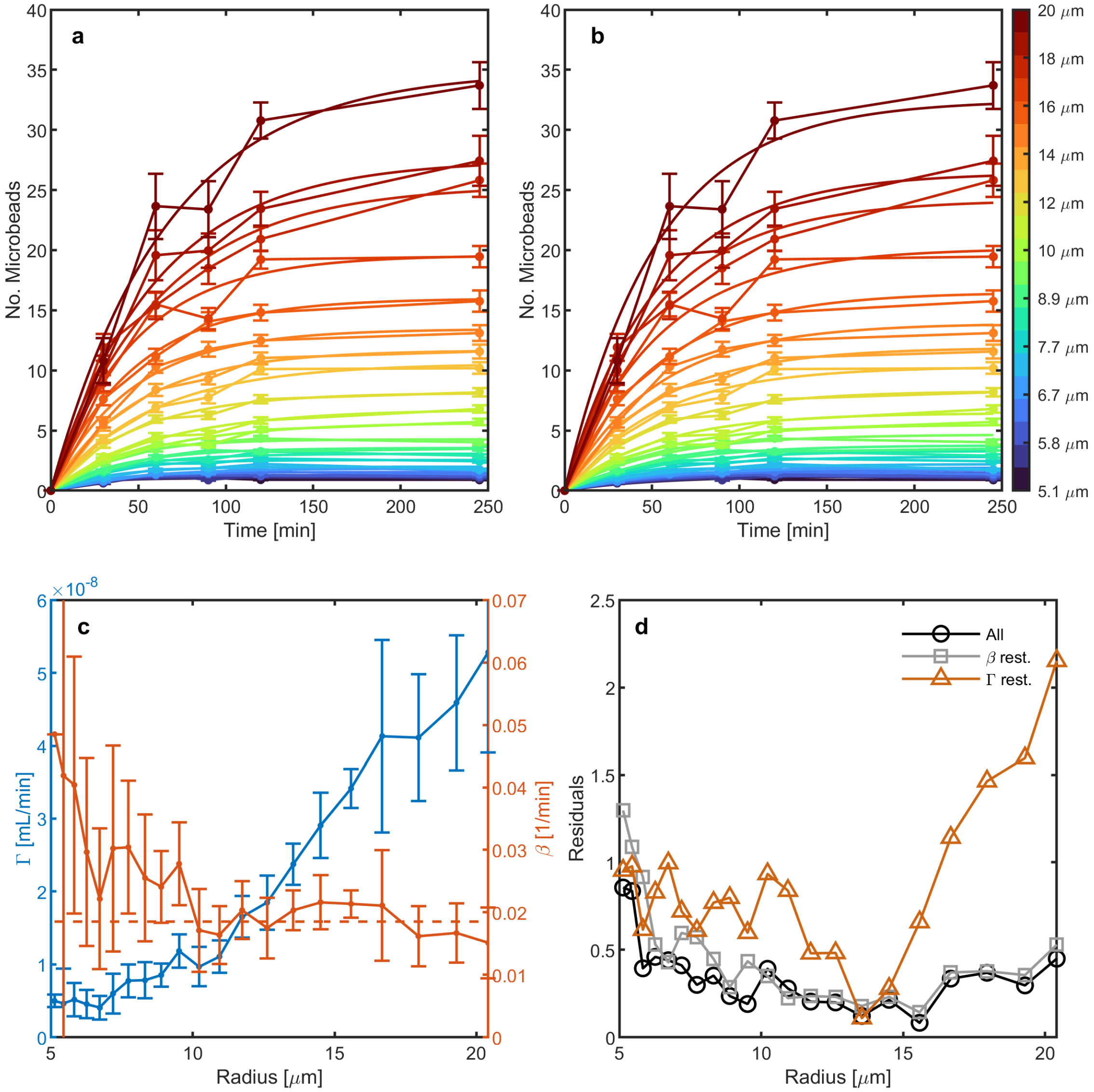

Figure S4: Average number of microbeads tracked over time for multicellular groups of various size classes. (a) Colors correspond to size bins. The line fits are regression from Equation 13, allowing both β and Γ to vary. Errorbars are one standard deviation. (b) Fits to the same data where β is held constant. (c) Fit parameters from (a) showing the size dependence of both Γ (blue, left axis) and β (red, right axis). The dashed horizontal line is the value $\bar{\beta} = 0.019\text{min}^{-1}$, used for fits in (b). Errorbars represent 2 standard errors in the fit value. (d) Sum of the root mean square of the residuals for each size class timetrack.

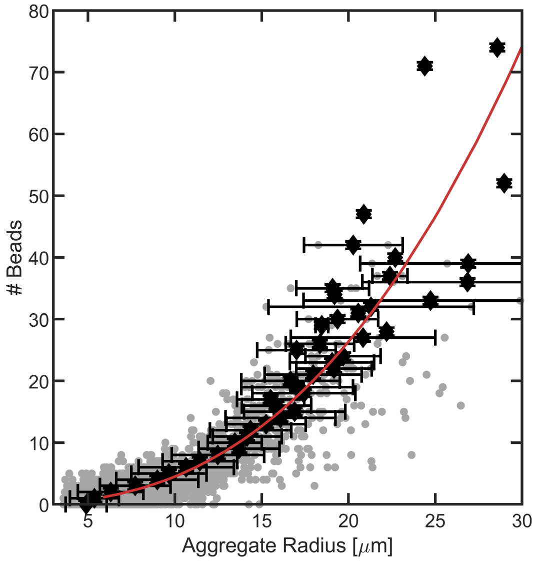

Figure S5: Number of microbeads attached per multicellular group as a function of multicellular group size Gray points are individual instances. Black diamonds represent avaverage number of beads for a size bin of a group radius. Horizontal error bars represent standard deviation for radii in the bin, vertical error bars are an estimated error from hand counting. Red line is a power law regression via equation 26. Data are replotted from Figure 2b and represent $N = 3150$ individual data points from one biological replicate.

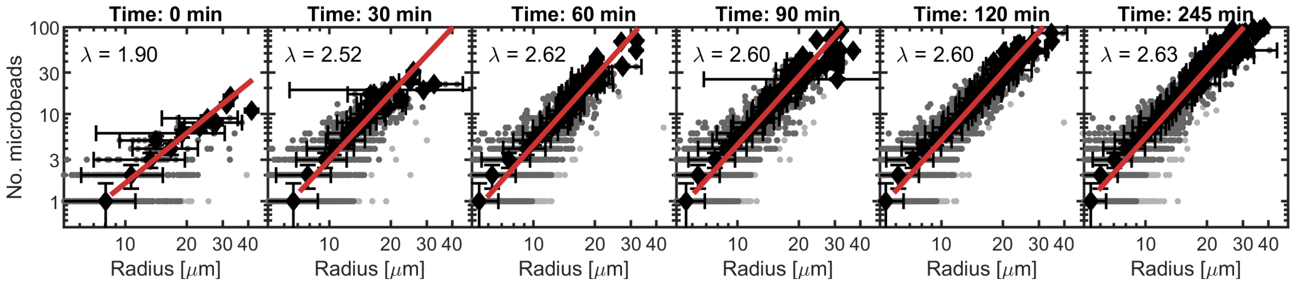

Figure S6: Number of microbeads attached per multicellular group as a function of multicellular group size sampled over time. Black diamonds represent averages in each bin. Horizontal error bars are the standard deviation for radii in the bin, vertical error bars are an estimated error in counting particles. Red line is a power law regression. The number of datapoints reported in each panel are: $N = \{1893, 2239, 3442, 3152, 3939, 6167\}$

in Figure S5.

3 Varying incubation, concentration, and shaking

3.1 Effect of incubation interval on empirical measurement of encounter kernel

In Figure S6, we show the number of microbeads attached to each multicellular group for each timepoint, as a function of the multicellular group size. We found that after about 60 minutes of incubation, there was relatively little change in the nature of power law fit exponent between timepoints, indicating that a steady-state scaling relationship had been reached.

3.2 Changing bead concentration

Our attachment/detachment model predicts that the concentration of beads has a linear relationship with the number of beads attached. To test this prediction, we incubated microbial aggregates with three different bead concentrations of $n_b = \{1 * 10^6, 5 * 10^6, 1 * 10^7\}$ beads/mL. We counted the number of beads attached per aggregate in the usual way. Then, we divided the number of beads attached per aggregate by the nominal bead concentration to obtain a measurement of the relative number of attached microbeads, which we label as P/C. In Figure S7 we show that the three different concentrations

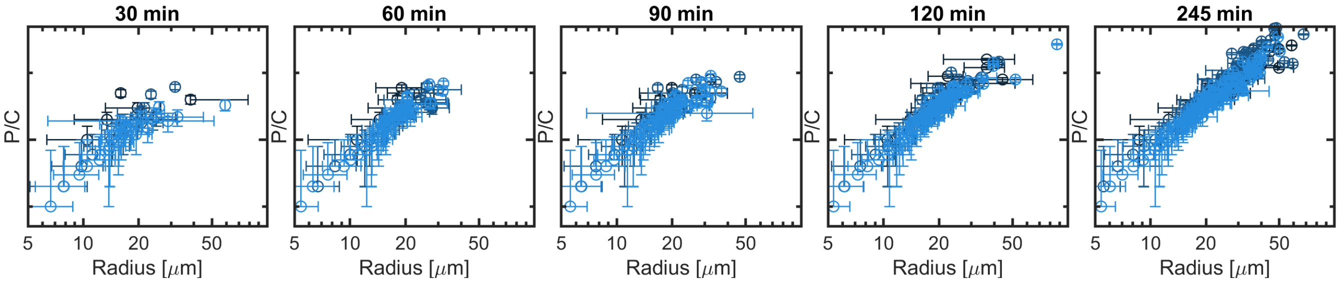

Figure S7: Number of microbeads attached per multicellular group per unit concentration as a function of group size sampled over time. P/C is the number of microbeads attached per group, divided by the nominal microbead concentration, for three different concentrations of microbeads. From darkest to lightest blue, nominal concentrations are $C = \{10^6, 5 * 10^6, 10^7\}$ beads per milliliter. For clarity, we show the average group radius for groups with P microbeads attached, with errorbars. Horizontal error bars are the standard deviation for radii in the bin, vertical error bars are an estimated error in counting microbeads. The number of groups shown in each panel for concentrations $\{10^6, 5 * 10^6, 10^7\}$ is as follows: $T = 30$min: $N = \{2042, 2025, 2239\}$, $T = 60$min: $N = \{2286, 3130, 3442\}$, $T = 90$min: $N = \{2663, 2868, 3152\}$, $T = 120$min: $N = \{2837, 2790, 3939\}$, $T = 245$min: $N = \{3422, 5136, 6167\}$.

collapse onto the same curve for all timepoints tested, as expected from our attachment/detachment model. This shows that for our experimentally chosen range of microbead concentrations, we live outside the effects from surface area competition and bead depletion, enabling us to measure explicitly geometric encounters alone.

3.3 Changing shaking properties

We wanted to understand how changing the turbulence of the fluid changes the phenomenological behavior we observed. We approached this in two separate ways. First, we changed the intensity at which we shook the co-culture of multicellular groups and beads, by changing the angular speed of the orbital shaker between 50 and 150 rpm. For each case, we counted the number of beads per multicellular group in the same manner as described above. In Figure S8, we show regressions generated from these three cases. Each were left to shake at their given intensity for 60 min before sampling. We found that increasing the shaking speed decreased the scaling exponent, as reported in the main text.

Second, we changed the shaker and/or beaker that we used to agitate the culture. As a control, we used a single well of a 24-well plate shaken at 100 rpm, as used for all other microbead experiments. Then, we tested an additional two types of incubation. First, we used a larger culturing flask, containing 15 mL of fluid total, and ran it on the same 100 rpm orbital shaker. Next, we changed the shaker to be a side to side platform shaker, which provides a slightly different type of turbulent environment. The side-to-side shaking speed was set to 107 rpm. We found that using different shakers generally led to different scaling exponents for the number of beads vs. aggregate size. This is expected, as the turbulent properties of a fluid are highly sensitive to the boundary conditions [12]. For example, the frictional area of contact between a fluid and its container changes significantly when filling flasks with different volumes and leads to differences in the energy dissipation rate.

4 Changing microbead surface chemistry

While so far, our goal has been to quantify geometric encounters between multicellular groups and particulate resources, we wondered whether using a different type of microbead surface chemistry would significantly alter our results. In general, particulate resources can have a wide range of surface chemistries that may affect attachment rates. Our hypothesis was that while geometric encounter rates would not be affected, the attachment efficiency would. To address this point directly, we performed additional experiments using amine-modified beads and compared the results to those obtained with carboxyl-modified beads.

We conducted time-resolved experiments using amine-modified beads at the same nominal concentration and timepoints as carboxyl-modified beads in parallel. Overall, we found that amine-modified beads exhibit scaling behavior that is very similar to that of carboxyl-modified beads (Supplemental Figure S10). The fitted scaling exponents for amine beads are slightly higher on average, but in most cases remain within the 95% confidence interval of the carboxyl bead measurements and are not statistically distinguishable.

To facilitate direct comparison, we overlaid the mean bead-attachment data for amine and carboxyl beads at each timepoint, along with their corresponding regressions (Supplemental Figure S10c). This analysis shows that, after correcting for bead concentration, both bead types fall within the same quantitative range and exhibit nearly identical scaling behavior.

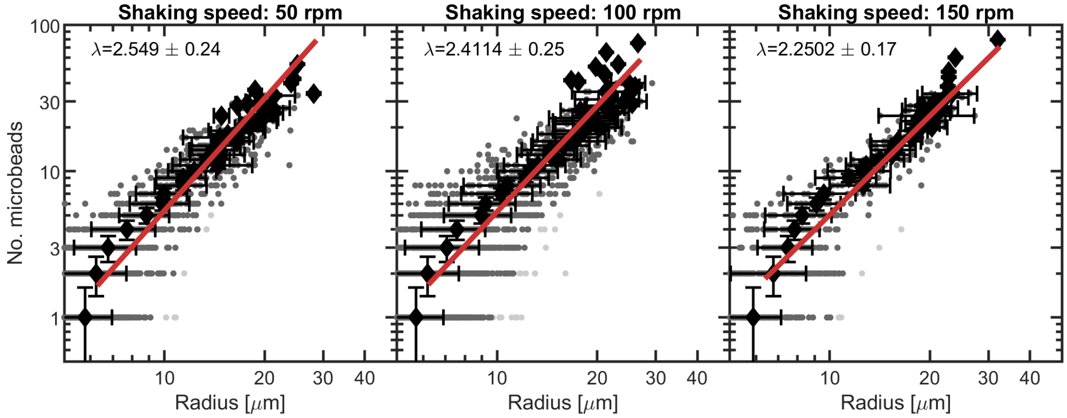

Figure S8: Distributions of microbeads per bacterial group, measured at different shaking speeds. All three shaking speeds are with an orbital rotation. Black diamonds represent averages in each bin. Horizontal error bars are the standard deviation for radii in the bin, vertical error bars are an estimated error in counting microbeads. Red line is a power-law regression, and the power λ is displayed in the panel. For the three panels, the number of groups counted was $N = \{1472, 2666, 781\}$.

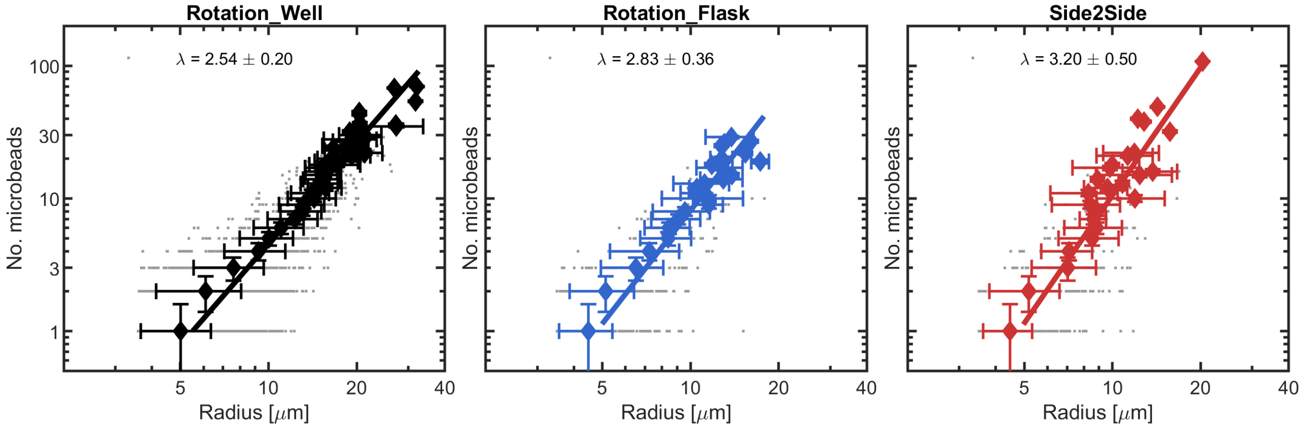

Figure S9: Distributions of microbeads per bacterial group, measured for different shaking paths (orbital vs. linear) and different culture chambers. Left: rotation well is an orbital shaker at 100 rpm and a 24-well plate with 0.5 mL of culture. Middle: rotation flask is an orbital shaker at 100 rpm and a 50 mL flask with 15 mL of culture. Right: Side to Side is a linear shaker at 107 rpm and a 24 well plate with 0.5 mL of culture. Diamonds represent averages in each bin. Horizontal error bars are the standard deviation for radii in the bin, vertical error bars are an estimated error in counting microbeads. Overlaid lines are a power-law regression, and the power λ is displayed in text in each panel. For the three panels, the number of groups counted was $N = [4189, 1594, 1250]$.

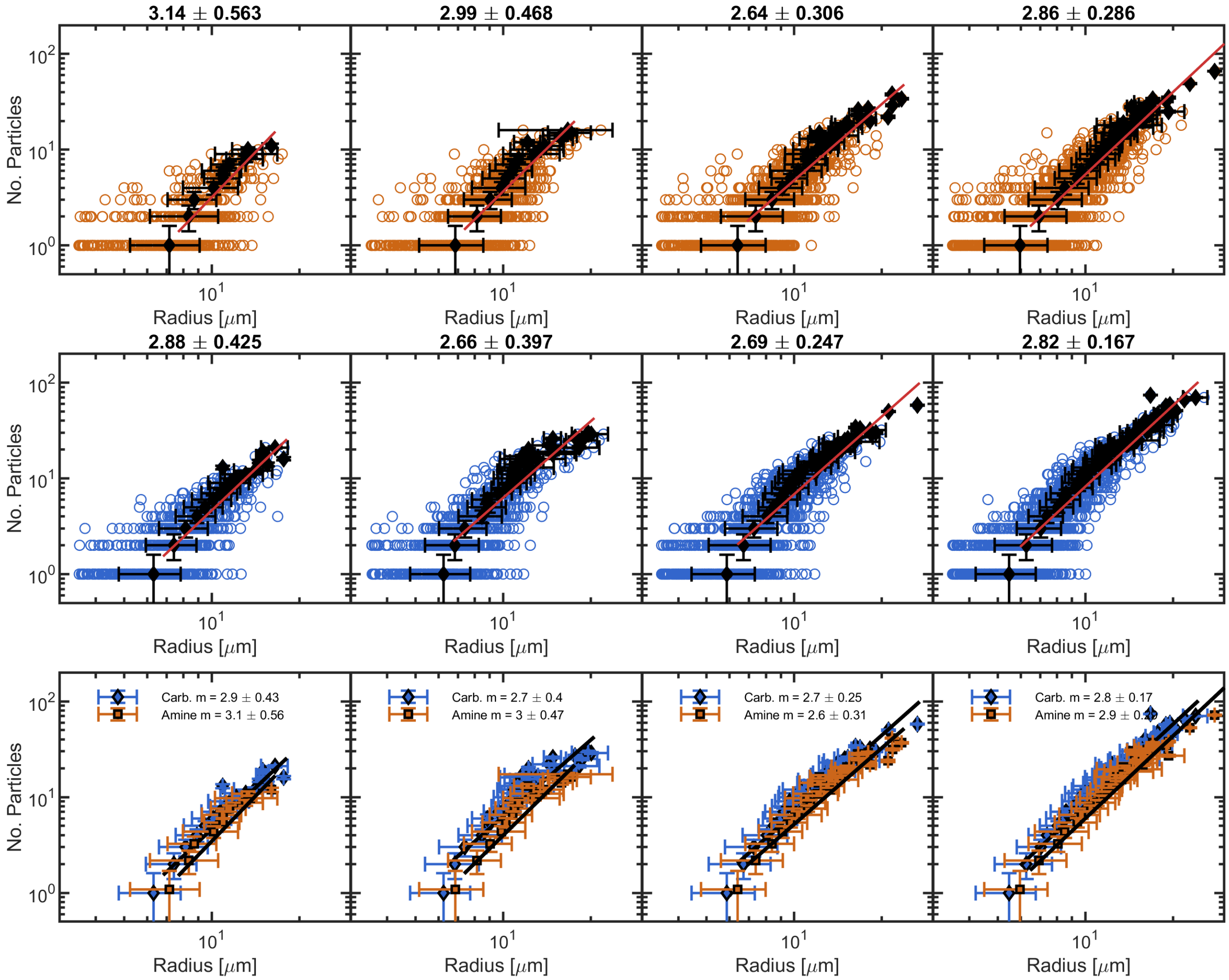

Figure S10: Microbead interactions for two different surface chemistries.

It appears that the groups attach to carboxyl-modified beads with a slightly higher attachment efficiency, as denoted by the different prefactors for the scaling law.

4.1 Experimental details

Briefly, 1 mL of an overnight culture (24 h; inoculum $\sim 10^3$ cells per mL) was added to each well of a 24-well plate, with three technical replicates per condition. Fluorescent beads (10^7 per mL, based on the manufacturer's nominal concentration) were added following 20 min of sonication. We found that the amine-modified beads were more difficult to disperse uniformly in artificial seawater; therefore,we first sonicated these beads in Milli-Q water and subsequently diluted into MBL seawater, which produced bead size distributions comparable to those of carboxyl-modified beads sonicated directly in MBL medium.

Plates were incubated at 25C with shaking at 100 rpm and sampled at 30, 60, 90, and 140 min. At each timepoint, 30 microliters was removed for imaging, allowed to settle for about 5 min, and imaged using tiled, z-stacked acquisitions ($\Delta z = 5$ microns) at 15x magnification. Identical imaging parameters were used across all conditions. Z-stacks were projected using Nikon's EDF algorithm prior to analysis.

Multicellular groups were segmented from DIC images using Ilastik as in all other experiments, while fluorescent beads were identified using a global threshold, again as in all other experiments. Bead counts per group were then plotted against group size for each replicate, timepoint, and bead type.

We observed that aggregates exposed to amine-modified beads exhibited a slightly lower overall bead attachment rate. To determine whether this difference arose from the differences in surface chemistry or from small differences in bead concentration, we quantified bead densities directly from representative image regions. This analysis revealed that amine-bead images contained approximately 92% of the bead density observed in carboxyl-bead images. After correcting for this concentration difference, the behaviors of amine- and carboxyl-modified beads were very close, with amine beads showing a marginally lower attachment efficiency (Supplemental Figure S10).

These additional experiments demonstrate that the observed scaling of encounter rates is robust to changes in bead surface chemistry. While surface chemistry may modestly affect attachment efficiency, it does not qualitatively alter the scaling behavior central to geometric encounters.

5 Connection between shaking and relevant physical conditions

To connect the shaking conditions of the wells to relevant ecological conditions, we make an order-of-magnitude estimate for the flow properties. First, we measure known properties of our experimental setup: the orbital shaker has a radius of orbit $a = 9.7mm$, measured by video of the shaking platform, it has an orbital angular frequency of $\omega = 100rpm = 10.5rad/s$, the wells have radius $b = 7.8mm$ (from VWR's printed specifications) and contain a volume of fluid, $v = 1mL$, and the water we take to have kinematic viscosity $\nu = 10^{-6}m^2/s$ and density $\rho = 10^3kg/m^3$.

We estimate the Reynold's number in two ways: from this paper of shaking in flasks [13], they use the approximation that $Re \approx \omega v^{2/3}/\nu$, which returns $Re \approx 1000$. A second way, would be to estimate the tangential velocity u, and use the well radius as a characteristic length scale, and then calculate $Re = ub/\nu$. For our measured values, this returns $Re = 1400$. Using the same approximations, we can estimate the Reynold's number of shaking in a larger flask (i.e. Supplemental Figure S9), with 15mL of fluid, to be $Re = 6000$. Both estimated Reynold's numbers lie in the transitional regime between fully developed isotropic turbulence ($Re \sim \mathcal{O}(10^4)$) [14], and highly ordered flow $Re < 100$. This magnitude Reynold's number, at this lengthscale, is common in marine systems. This can be intuited by estimating the necessary velocity of water flow for a 1 centimeter characteristic length scale: $u = Re * \nu/L$, which returns $u \approx 10$ cm/s. This kind of velocity is recorded in tidal zones [15], sheltered coastal waters [16, 17], and even recent deep sea measurements [18].

6 Poisson distribution fits

We fit a Poisson distribution to the distribution of the number of microbeads attached to multicellular groups in various size classes. We did this by binning multicellular groups into logarithmically-spaced size bins. For each size bin, we generated a histogram of the number of multicellular groups with p microbeads attached. We normalized this histogram to produce a probability density function (PDF): each bin was normalized like $v_i = c_i/(N * w_i)$, where c_i is the number of counts in the bin, $w_i = 1$ is the width of the bin, and $N = \sum_i c_i$ is the total number of multicellular groups counted. We then produced a predicted PDF from a Poisson distribution with the same mean, $f(k) = \bar{p}^k e^{-\bar{p}}/k!$, where $\bar{p}$ is the average number of beads attached per multicellular aggregate. We overlay a full panel of these histograms and the Poisson predictions in Figure S11.

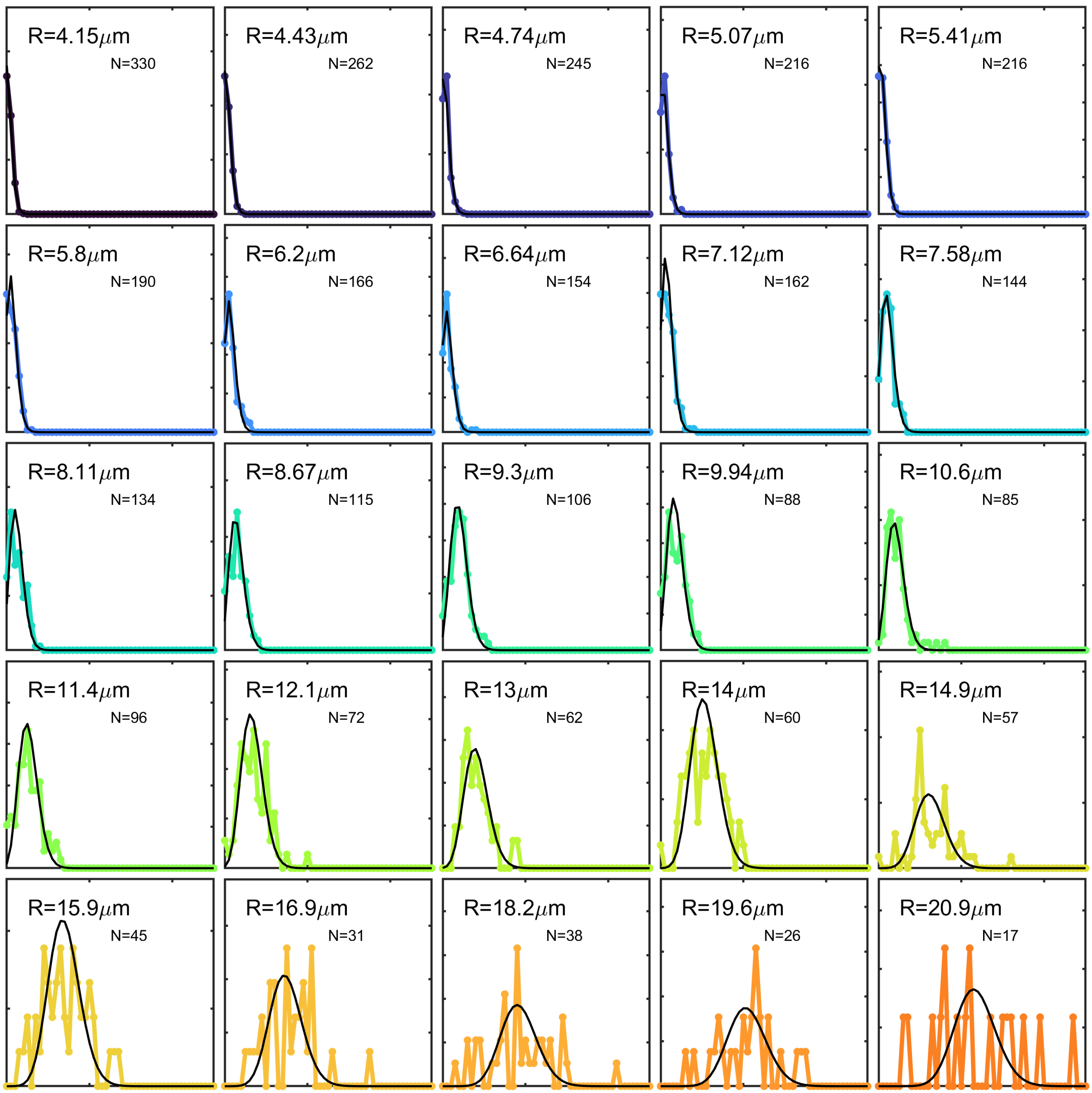

Figure S11: Poisson distribution matches microbead number histograms for a wide range of multicellular group sizes. Probability density functions of the number of microbeads attached per multicellular group for groups in different size classes, overlaid with Poisson distribution expectations. The x-axis ranges from 0 to 50 μm in each panel. The y-axis lower limit is always 0, the upper limit is variable to capture the entire distribution. Data are replotted from Figure 2. Text in each panel indicates the average radius of the groups in each bin R, and the number of groups in each histogram N. We only show bins where $N > 10$.

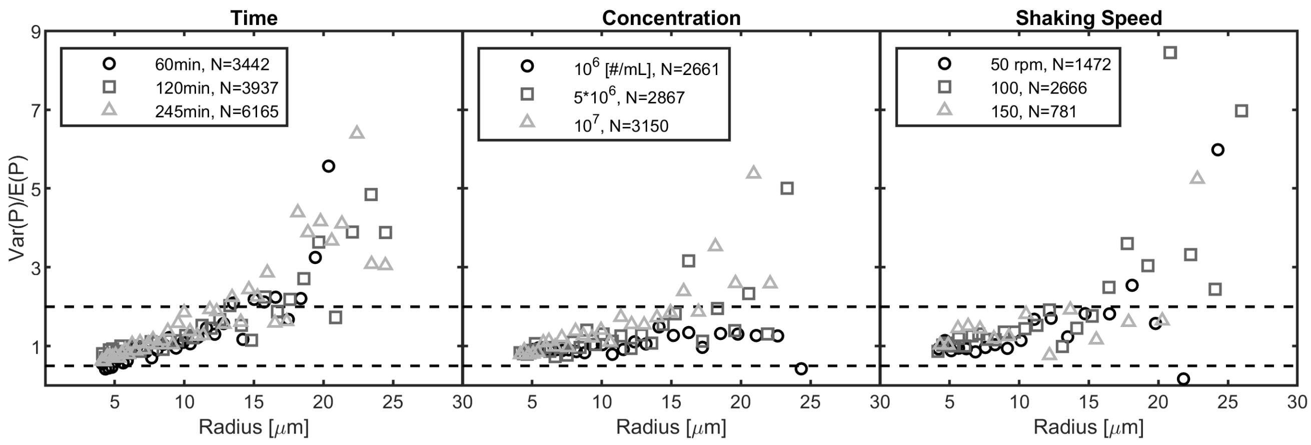

Figure S12: Level of agreement with Poisson predictions for a wide range of treatments. Plotted is the variance in the number of microbeads attached per group, divided by the mean number of microbeads attached per group, ($s = \sigma^2/\langle P \rangle$) after binning groups by size. The region between the horizontal dashed lines is where $0.5 < s < 2$. Left: 3 different timepoints, where the concentration of microbeads was 10^7 beads per mL. Center: 3 different concentrations of bead incubations, all sampled at $T = 90$min. Right: 3 different shaking speeds on an orbital shaker after 60min incubation with 10^7mL^{-1} bead concentration. Legends indicate the total number of groups counted in each treatment.

6.1 Measuring agreement with and deviation from Poisson predictions

We measured the degree of agreement with Poisson predictions by comparing the measured mean number of microbeads to the variance in the number of microbeads for binned size classes. We created a test statistic to quantify this comparison,

$$s(r) = \langle (P - \langle P \rangle)^2 \rangle / \langle P \rangle \tag{22}$$

where $\langle . \rangle$ indicates an average over all the multicellular groups in the size bin given by the multicellular group radius r, and P is the random variable of the number of attached microbeads to each multicellular group. Since a characteristic of the Poisson distribution is that the mean and variance have equal values, i.e. $s = 1$, we labeled values of the test statistic that were in the range $0.5 < s < 2$ to agree with the Poisson prediction, and values outside this range to disagree. We found that, for a broad range of incubation times, concentrations, and shaking speeds, the test statistic behaved qualitatively the same (Figure S12). Smaller multicellular groups had low values of s, which increased as multicellular groups became larger. The exact size at which s generally exceeded our defined range varied slightly between treatments.

6.2 Testing low statistical power of various size bins

Because the most significant deviations from the Poisson predictions tended to come from size bins where there were relatively few multicellular groups, we wondered if the discrepancy was caused by low statistical counts. To test this, we first ran a random sampling simulation *in silico*, to test how often a Poisson-distributed sample with m members would fail our test statistic criterion, $0.5 < s = \sigma^2/\mu < 2$. We did this by sampling m instances randomly from a Poisson probability density distribution with mean $\lambda = 10$. We then calculated the sample mean, $\bar{x} = \langle x \rangle = 1/m \sum_{i=1}^{m} x_i$, and sample variance, $\sigma^2 = \langle (x - \bar{x})^2 \rangle$, of the set of m instances. We used these sample statistics to calculate our test statistic, $s = \sigma^2/\bar{x}$. We found that samples of size $m = 2$ failed to adhere to the range $0.5, s < 2$ up to 68% of the time. However, when sampling $m = 10$, this only failed our statistic on average about 14% of the time. By comparison, the fail rate of any size bins within our dataset containing between 2 and 10 members was 86%, indicating that random sampling is not the cause of the discrepancy.

6.3 Connecting multicellular group shape with elevated variance in microbead encounters

We were interested in determining why large multicellular groups generally have a larger variance in the number of microbeads attached to them than the Poisson distribution predicts for their mean number of attachments. We can group reasons for this discrepancy into two general classes: (a) there is a biological cause - for example, it is possible that larger multicellular groups have more variance in their adhesiveness to the microbeads - or (b) there is a physical cause.

One possibility for the physical cause of this variance discrepancy is that large multicellular groups can deviate from spherical shapes. Deviations from sphericity can occur either through growth of the cells within the multicellular group, as cells are oblong and divide via binary fission, or they might occur because multicellular groups can encounter and interact with/adhere to each other. Especially at larger size, multicellular groups are likely to no longer be clonal. As multicellular

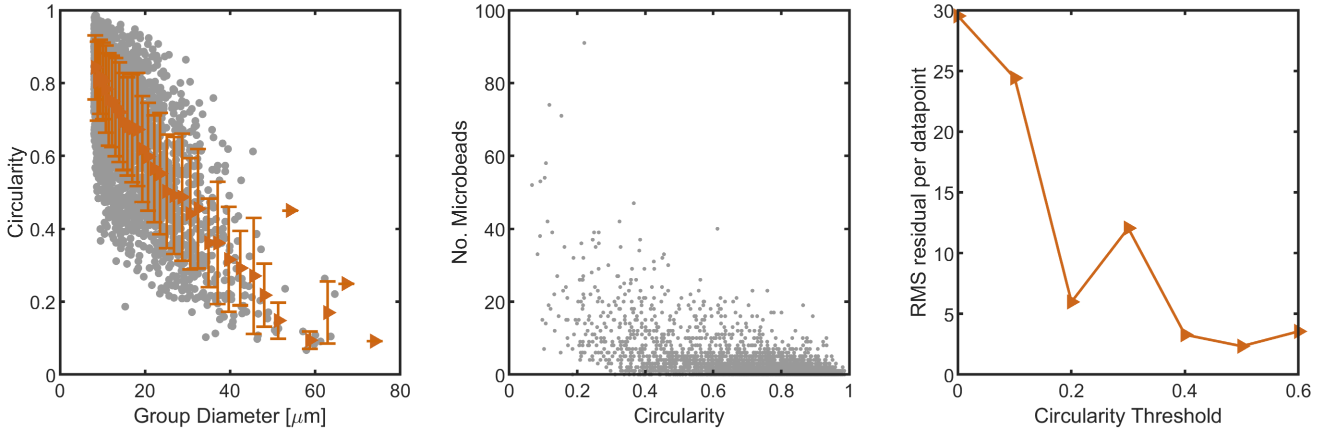

Figure S13: Loss of circularity in shape is a contributor to elevated variance in microbead attachments. (a) Circularity decreases with multicellular group size. Gray points are individual measurements ($N = 3150$), orange triangles with errorbars are averages within size bins with standard deviations in circularity. (b) The number of microbeads attached per group vs. the circularity of the group. Gray points are individual group measurements, replotted from S10a. (c) The root-mean-square residual, $r_{rms} = \sqrt{\langle(\bar{p}_i - \sigma_i^2)^2\rangle_i}$, vs. a circularity threshold C^* removing all individuals with circularity less than C^*. All data is from the same experiment as Figure 2b.

groups grow in size and concentration, the likelihood that they encounter and attach to one another increases. Such aggregated groups can exhibit strong deviations from sphericity. For example, two spherical multicellular groups, each 10 µm in radius, stuck together such that their centroids are separated by 10 µm, will have a cross-section circularity of 0.69. As multicellular groups adhere into larger aggregates, there become many possible shapes that deviate from sphericity; in other words, for sphericities < 1, there are increasingly many possible shapes. As physical encounters are affected by geometry, either a large spread in sphericity, or a simple decrease in sphericity, could contribute to a larger spread in physical encounters. We hypothesized that this physical variance could be contributing to the deviations from the Poisson expectations that we observed for larger group sizes.

To test this hypothesis, we measured the circularity of our groups for one case (we chose $n_b = 1 * 10^7$ beads/mL, $\Delta t = 90$ min, and shaking speed 100 rpm). We found a strong negative correlation between multicellular group size and circularity (Figure S13, supporting the idea that there is more variance in physical shape at larger sizes. We also found that the spread in circularity increased as aggregate diameter increased. Next, we found that the most aspherical groups exhibited the most variance in the number of beads attached. We then applied a range of circularity thresholds to the dataset, whereby we filtered out any groups with cross-sectional circularity below c. For each circularity threshold, we measured the size-dependent mean number of attached microbeads $\bar{p}$ and the size-dependent variance σ^2 and calculated the root-mean-square residual between the mean and variance in each size class ($r_{rms} = \sqrt{\langle(\bar{p}_i - \sigma_i^2)^2\rangle}$). We found that the mean and variance became more similar with more severe circularity thresholds, supporting our hypothesis that deviations from circularity play a contributing role in the elevated variance as compared to the mean rate of attachments for large multicellular groups.

7 Measuring number of cells per group

Cellular packing within individual groups was measured for 10 multicellular groups via confocal microscopy. We used cell stain Syto-9 [Invitrogen] at ∼5 µM concentration to label individual cells within 12B01 multicellular group grown on alginate for 36 hrs. Then, we used a Leica point-scanning confocal microscope (see Methods) to image cells within groups. We used a custom segmentation algorithm to segment individual cells within groups. Example segmentation is displayed in Supplemental Figure S14.

7.1 A description of the 3D segmentation algorithm

In order to segment and count the number of cells in the 3D confocal images, we defined an algorithm that we describe here. It proceeds in parts: Part 0 - Pre-processing, Part 1 - Binarization, Part 2 - Watershedding, Part 3 - Creating Isosurfaces, and Part 4 - Ellipsoid Fitting.

Part 0 - Pre-processing. In the pre-processing step, our goal was to remove extraneous objects (nearby groups and cells not attached to the group of interest) from the scene. First, we blurred the image with an 11x11 gaussian filter with $\sigma = 5$ pixels, followed by a lenient block threshold to binarize the entire 3D image. This ensured that a single group in an image was blurred enough to be counted as one object. We followed this binarization with a round of cubic mask dilation (3 pixels),

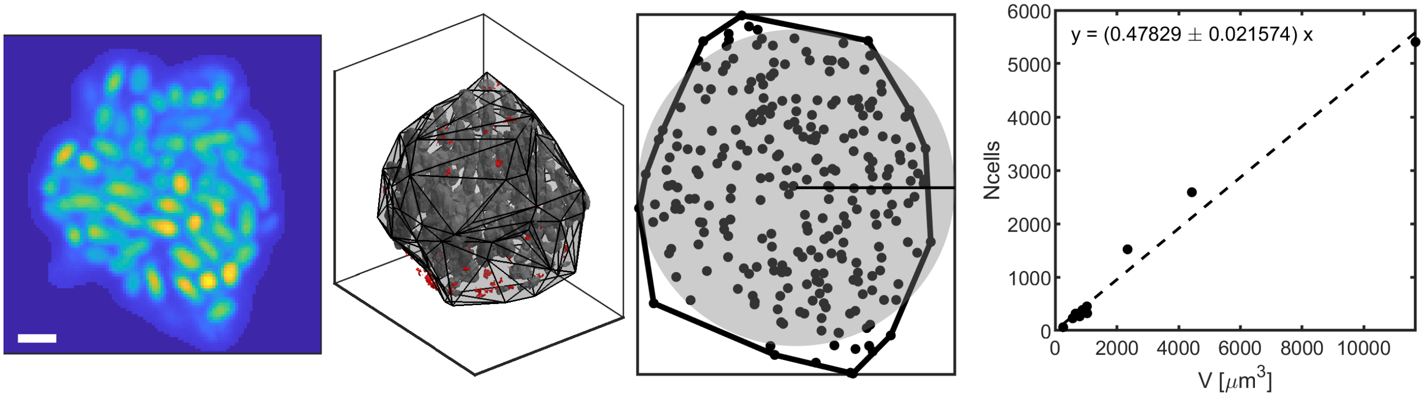

Figure S14: Cell count is linearly correlated with multicellular group size. (a) Example confocal z-slice of one bacterial group labeled with Syto-9 fluorescent stain. Scalebar is 2μm. (b) A 3D automated cell segmentation algorithm segmented the cells in each multicellular group. Gray surfaces are segmented cells. In red are shown candidate cells whose ellipsoid fits recorded either negative volumes, negative cell aspect ratios, or aspect ratios above 10. These surfaces were removed from further consideration. (c) Cell centers were projected onto a single z-plane. The 2D convex hull of the points is displayed overtop. A disk of the same cross-sectional area as the convex hull is displayed in gray. The radius of that disk was used to estimate volume. (d) The number of cells is plotted vs. multicellular group volume, showing a linear relationship with $r^2 = 0.98$.

followed by a filling algorithm to fill any extra gaps that had not been caught by the processing so far. We then found all connected components in the mask, and kept only the largest one. We then used this mask to clear any extraneous objects (other cells, smaller groups) from the original image.

Part 1 - Binarization. Next, we sought to binarize individual cells from the background. Since cells become dimmer farther away from the coverslip, we used a local thresholding method known as the Niblack method to binarize each z-slice separately. This method involves computing the average value of a neighborhood surrounding a pixel of interest, and also the standard deviation of the pixels around the pixel of interest. Then, if the pixel of interest exceeds the mean value of the neighborhood by a user-chosen fraction of the standard deviation, the pixel is considered a foreground object. We used a neighborhood of sizes 15x15 pixels, and $\sigma_{\text{thresh}} = 0.5$.

Part 2 - Watershedding. The output from Part 1 is a 3D mask of pixels. However, it is not always able to completely segment neighboring cells from one another. To accomplish this, we used a 3D watershed algorithm that took into account both the local pixel value and its distance to the nearest background pixel to set the watershed lines. We then turned all pixels along the watershed lines into background pixels, effectively splitting the image along these contours. Within each watershed basin, there was thus a single connected component, where all pixels of the object are value 1, and the local background, which has pixels of value 0.

Part 3 - We next determined the surface of each connected component as an isosurface with value 0.5, and listed the vertices of this surface for the next step.

Part 4 - As the bacterial cells are convex objects that can be modeled as ellipsoids, we fit ellipsoids to each connected component vertex list generated from the determined isosurfaces. We later used these ellipsoid fits to measure cellular volume and aspect ratio, and excluded connected components with extreme values for these measurements from the remaining analysis. The parameters we defined as extreme included any negative volume, negative aspect ratio, and any aspect ratio greater than 10. Finally, we saved the information of isosurfaces and ellipsoid fits to file for later use.

Once segmentations were complete, we counted the number of cells in each group and measured the group size. To most accurately emulate how this was measured during brightfield experiments, we first projected all cell centers onto a single z plane, then found the 2D convex hull polygon surrounding these points, then found the radius of a circle with the same cross-sectional area as this convex hull. This radius was then used to obtain an estimated volume of an equivalent sphere. We then used these measurements to generate a regression for the number of cells in a group vs. its volume (Figure S14). We found that the relationship between number of cells and volume was linear: a log-log regression found that the power-law slope was $a = 1.004 \pm 0.04$ (1 sigma). A linear regression returned the fit relationship to be: $N = 0.48V + 24.1$, and had a Pearson's correlation coefficient of $r^2 = 0.993$. We used this regression relationship to estimate the number of cells in all groups henceforth.

8 Cell state competition simulations

To simulate competing populations using different strategies to obtain resources from a common pool, we simulated a population of individuals that can grow, divide and die. Inspired by experiments, where multicellular groups are only

transient and eventually burst, we assigned individuals to one of two competing cell states. All individuals, no matter their "state", start at the size of a single cell. There is no flux of individuals between the two states. We always seeded competition simulations as occurring between a population of $N = 1000$ cells in state S1, and $N = 1000$ cells in state S2.

Growth and decay In both states, individuals grow in size based on encountering food, and reduce in size if they do not encounter enough food. Each individual's biomass changes like $b_i(t + \Delta t) = (\mu_i - \delta)b_i(t)$ where μ_i represents the growth according to the amount of resources accrued, δ represents a decay rate, and $i \in [1, N]$ enumerates the individual. All individuals start with a biomass of b_0. In State 1, which can be considered the "single-celled state", if an individual's biomass reaches $2.0b_0$, it divides into two individuals of equal biomass (each being b_0). In State 2, which can be considered the "multicellular state", individuals never divide, instead continuing to grow in size indefinitely. For either state, if the individual's biomass reaches $0.95b_0$, then it is removed from the population.

Assigning growth rates We consider a Monod growth model for growth and division [19]. In this model, growth rate is a saturating function of the amount of food per cell encountered. In other words, the biomass of an individual i increases with rate

$$\mu_i = \frac{\mu_{max}\gamma_i}{\gamma_i + K_m} \tag{23}$$

where μ_{max} is the maximum possible growth rate, with corresponding doubling time $\tau_m = 1/\mu_{max}$, K_m is the Monod half-saturation constant (which we hold constant between states, and set to be equal to receiving one particle of food per doubling time, τ_m), and $\gamma_i = E_i/N_i$ is the per-capita number of food particles encountered during the minimum doubling time τ_m.

Fluctuations in food We ran two separate methods for assigning food, either with or without fluctuations in the amount of food received. Without fluctuations, the amount of food received by each individual is exactly the expected value, being given by $\langle\gamma_i\rangle = \langle E_i\rangle/N_i$ (see below), and can be fractional. With fluctuations, the amount of food received by each individual is randomly assigned from a Poisson distribution with mean and variance given by $\langle E_i\rangle$, and must be an integer value (i.e. the food is discretized into particles).

Assigning Death Rates In this model, we held the decay rate δ constant between the states, at a rate of 0.03 units per doubling time.

Calculating the number of resource encounters The general encounter kernel is

$$\Gamma = \Gamma_D + \Gamma_T + \Gamma_B + ... \tag{24}$$

where the different terms represent the contributions from diffusion, turbulent flow, bouyancy, and more. The number of encounters with food particles during a time period Δt that an individual can expect is given by

$$\langle E\rangle = \Gamma c_0 \Delta t \tag{25}$$

where c_0 is the concentration of the food particles, and the brackets $\langle ...\rangle$ denote an ensemble average.

Using our empirical measurements of the turbulence kernel, we write the expected number of encounters during a time Δt:

$$\langle E_i\rangle = Ac_0\Delta t(\theta_i + \theta_0)^\lambda \tag{26}$$

where A is a constant that depends only on properties of the fluid, like the energy dissipation rate and viscosity, and θ_0 is the size of the food particles. The power law constant λ was empirically determined to reside between 2 and 3 in our prior experiments; we therefore used this range for λ in the remaining simulations. The constant $G_0 = Ac_0$ is the baseline rate of particle encounters; in other words, this is the basal rate of encounters per doubling time of the fastest doubling in the S1 state.

Running the competition We initialized a competition experiment with equal proportions of cells in State 1 and State 2. The competition consisted of rounds of selection. Each round, every individual was assigned food according to the food assignment protocol described above. From that, each individual's expected growth rate was calculated. The decay rate was subtracted from this expected growth rate, and the individual's biomass was changed accordingly. Finally, updates in cell division and death were recorded, and the simulation continued.

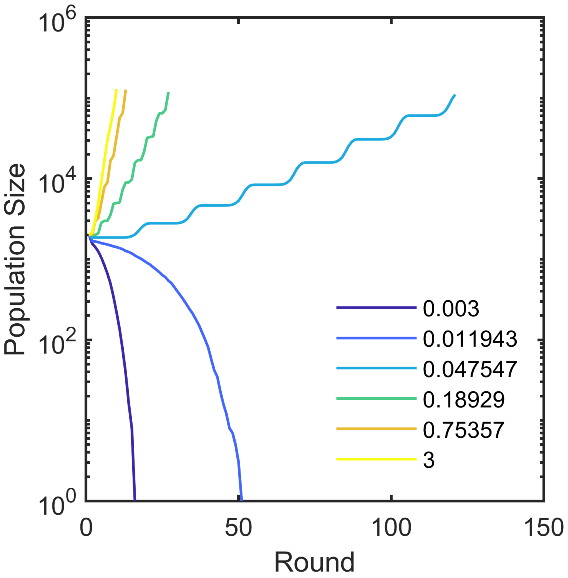

Figure S15: Population size over simulation time for 6 different simulations, each without any fluctuations in number of resources encountered. Six different concentrations of resources were chosen according to the average number of particles encountered per maximum cell doubling rate. Concentrations are shown in different colors, from very low concentration (equating to .003 number of encounters per fastest doubling period of the S1 state) to high concentration (3 encounters per basal doubling period of the S1 state).

Parameter sweeps We consider the scenario where there is a constant flux of food particles into the volume of interest for the competition. The concentration of food particles sets the baseline rate of encounters between individuals and resources. We chose to sweep resource concentrations such that individuals acquire relatively few resources per their fastest possible doubling time, τ_m; in this regime, cell growth is therefore limited by resource acquisition, especially since the two different strategies S1 and S2 share the same same max doubling rate. We normalized the resource encounters to occur per doubling time τ_m. The explicit range of concentrations we chose swept from between $0.03 \leq G_0 \leq 3$ for a single cell. We chose this range as this was where cells could actually grow. When we pushed the concentration below this level, the population died off (Supplemental Figure S15).

Including cell swimming We next sought to include the effect of cell swimming. We model cell swimming as a run-and-tumble pattern without chemotaxis. The nature of this type of swimming is very similar to pure diffusion, with an encounter kernel:

$$\Gamma_S = 4\pi(D_i + D_0)(\theta_i + \theta_0) \tag{27}$$

where D_0 is the thermal diffusion constant for a non-swimming particle, and D_i is the "effective" diffusion constant resulting from run-and-tumble motion. A higher D_i means that the cell is swimming more vigorously. We consider the scenario where $D_i \gg D_0$, and so D_0 can be considered neglible. Including this swimming term results in the form:

$$\langle E_i \rangle = Ac_0\Delta t(\theta_i + \theta_0)^\lambda + 4\pi c_0 \Delta t D_i(\theta_i + \theta_0) \tag{28}$$

which we used to assign expected encounters in simulation. We only allowed individuals (of either state) with biomass less than $2b_0$ to swim.

It is important to note that this simple addition of the effective diffusion term is only valid in the limits where there is no motility to the original encounter kernel, and where cell swimming is uncoupled to the other physical processes. In the case of coupling, this term would become significantly more complicated. Coupling between swimming and flow has been observed in other work [7], where they found that it arises from the bacterial shape. When bacteria are shaped like rods, they tend to line up with the flow more often than transverse to it. In the case of either spherical cells, or frequent and random flow changes, then we would expect this effect to be negligible in magnitude.

We swept swimming speeds with effective diffusion constants in the range $0.01 \leq D_1 \leq 3000$ μm^2/s, thereby sweeping from a range where swimming plays essentially no role in the rate of encounters, to where it plays a dominant role. We show the results from this sweep in Figure 5 of the main text.

To understand what these effective diffusion constants mean for a single swimming organism, we plot the relationship between swimming speed, characteristic run time before re-orientation, and the effective diffusion constant from the random

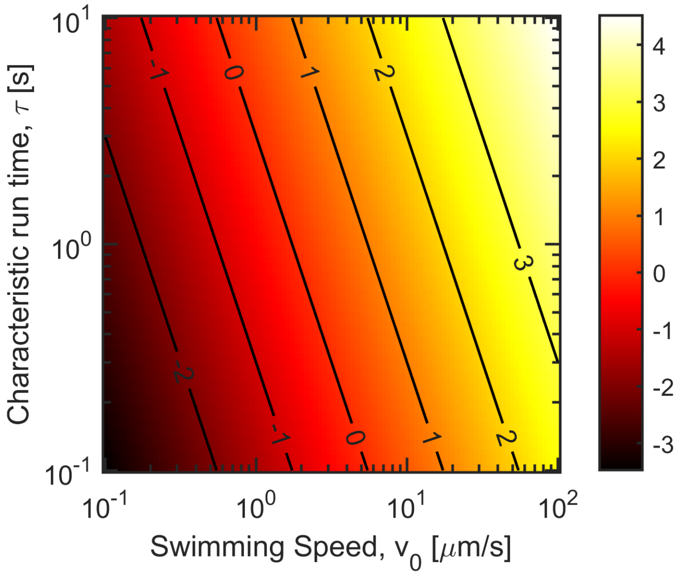

Figure S16: Effective diffusion constant from swimming. Color map represents the log of the diffusion constant from 10^{-3} to 10^{4} µm^2/s. Diffusion data are plotted as a function of the swimming speed and the characteristic run time, shown in log10. The labeled contours show swimming strengths of 10^{-2} through 10^{3} µm^2/s, indicating the range swept in simulations.

walk in Figure S16. This relationship is well-known [20], as $D = v^2\tau/3$, where $\tau = \sigma/(1-\phi)$, σ being the characteristic time between re-orientations, and ϕ being the characteristic angle cosine between runs. Here, we use $\phi = 0$, in other words, no correlation between the direction of subsequent runs.

Growth rate penalties We next investigated how strong any growth penalties for the multicellular state would have to be in order to recover an advantage for the single celled strategy. We did this by altering the maximum growth rate in the Monod kinetics, μ_{max}. We assigned a separate μ_{max} to each of the two cell states (i.e. μ_1 and μ_2), and varied their ratio, such that the fraction μ_2/μ_1 ranged from 0 (maximum penalty for being in the multicellular state) to 1 (no penalty). We show these results in Figure 5 of the main text.

8.1 Including toxic, phage-like particles

We extended our above simulation to understand if multicellular states can arise in a population where encounters with both food and toxic particles are governed by our empirical encounter kernel. What are the necessary ingredients that kill the ability for multicellular states to arise and coexist with single-cell states? Our hypothesis was that both the toxic particle concentration and the toxic particle "behavior" (e.g. the burst size) would affect the ability for multicellular states to arise.

We therefore added a new type of particle encounter in our simulations, that was sampled in the same way as the food encounters. Toxic particle encounters kill the individual with probability q, which we set $q = 1$ for all simulations. If there was ever a case where an individual encountered more than 1 toxic particle in a timestep, the individual was still killed only once. Food and toxic particle concentration was swept by altering the baseline encounter rate per doubling time, as above. Finally, we swept the burst size of the toxic particles - i.e. the number of new toxic particles that are generated from each cell death event, reminiscent of phage behavior. Each timestep, we calculated the net number of particles that were generated via $\Delta N = \sum_i BS * b(i)$, where $b(i)$ is the biomass of group i, BS is the burst size which was a constant number, and the sum over i is over groups infected within the last timestep. To simulate how bursts increased the global concentration of phage, we used a regression from our microbeads data to estimate the prefactor of the encounter kernel. With this known prefactor, and known microbead concentration, we therefore estimated the baseline rate of encounters. At each time step, we then updated this baseline rate of encounters according to the increase in global phage concentration. In our simulations, there is no other phage flux, i.e. phage never die or are otherwise removed from the surrounding water. Therefore, in the long time limit, state 2 will always go extinct, as they will always eventually encounter a phage, and there is no mechanism to reproduce. We do not observe this extinction in some cases, because we restricted the simulation to 218 rounds (72 hours) maximum to restrict computing time to a reasonable limit.

Each simulation was seeded with 100 individuals in state 1 (single-celled) and 100 individuals in state 2 (multicelled), each individual having an initial biomass drawn from a Gaussian distribution with mean and variance. In order to reduce computation time, we set a cap for the maximum size of the population to be $\kappa = 10^5$ individuals. We ran the simulation until either state 2 went extinct, state 2 fixed in the population, κ was reached or until 218 rounds of time steps had occurred,

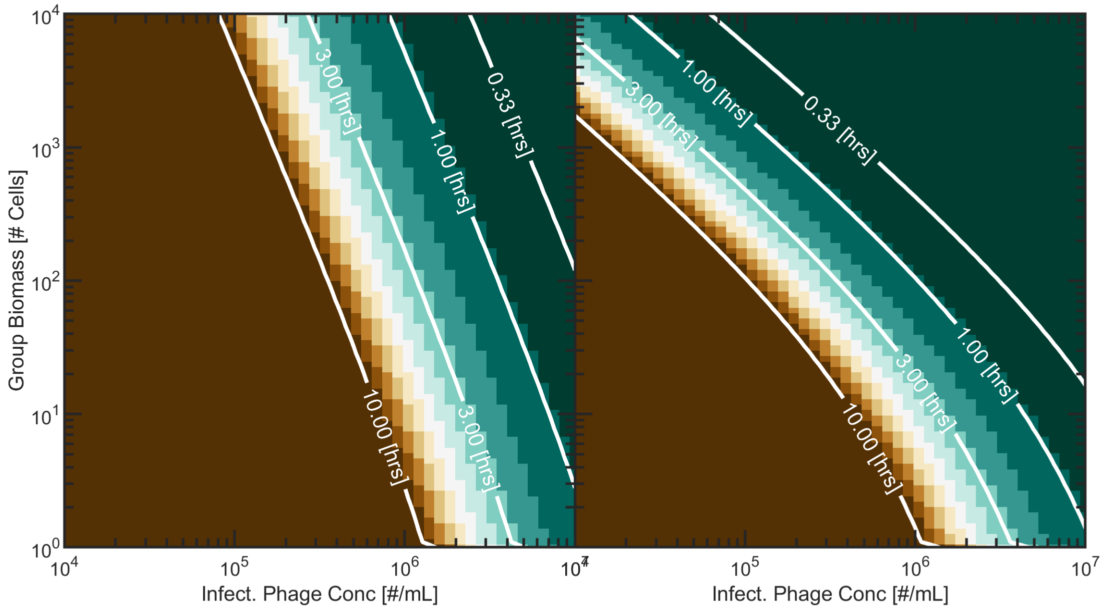

Figure S17: Expected timescale before infection Left: Diffusion alone drives encounters between microbial groups of different sizes, and phage 0.2 microns in diameter with varying concentration. Right: empircial encounter kernel with $\lambda = 2.8$.

which is akin to 72 hours if the maximum doubling rate is $1/20$ mins^{-1}.

We held the food concentration to be constant between all plotted simulations, akin to 0.1 encounters per fastest doubling time. We varied the burst size of the toxic particles according to 0, 50, 100, 200 PFUs per burst, which are values associated with burst sizes in the literature. We varied the phage concentration to sweep between 1000 times less common than food encounters, to just as common.

9 Theory of livable timescales

We calculated the expected encounter rates between groups and phage given only group size and phage concentration to understand the timescale over which groups can be expected to assemble, exist, and disperse. We call this timescale τ_L, or the livable timescale. First, we calculated the expected encounter kernel from diffusion. Second, we calculated the empirical encounter kernel that we derived in the rest of the paper. We show both encounter kernels separately in Supplemental Figure S17. We also added this encounter kernels via linear superposition; that plot is shown in the main text.

10 Gillespie simulations

We wished to calculate the livable timescale for groups of particular sizes, and take into account each individual encounter, dynamically changing the phage concentration after each event. We also wished to sweep burst size, phage concentration, and group size explicitly, rather than implicitly by varying the baseline rate of encounters. To accomplish this task, we turned to a Gillespie simulation scheme, which is well-known for modeling stochastic processes like biochemical reactions.

This simulation model allows us to explicitly probe encounters with particles of varying concentrations and via different physical processes more rigorously. We purposely do not consider bacterial cell growth in this model, as careful measurements of rates, saturation constants, burst sizes, incubation times, and more would be necessary to adequately model this system. An extreme real-world example of this would be if all metabolism of the cells goes into a process other than cell division. We first considered homogeneous populations where each individual is modeled as a sphere with N cells, so that the biomass of the group is related to the volume via the packing fraction, like $\phi = N v_c / V$, where v_c is the volume of a single cell and V is the net group volume. All phage particles have a diameter of 0.2 microns. We used the empirical encounter kernel from our microbeads experiments to model encounters.

The Gillespie algorithm works in the following way. First, an expected encounter rate with phage is calculated for each individual in the population. We add all those rates together, to form a net reaction rate $R = \sum_i = 1^M r_i$, where M is the number of individuals in the population. We sample a timestep Δt from an exponential distribution with mean $1/R$. This timestep is the random time between successive "reactions" - aka infections. We track the cumulative time of the simulation by addition $t_k = t_{k-1} + \Delta t_k$. We next assign which individual is suffering the reaction by randomly sampling the interval

$[0, 1]$, which has been subdivided into M equal pieces. When the random number a, drawn from the uniform distribution, lies in the range associated with individual i, this individual is immediately killed with probability q (which we set to be $q = 1$), the time t_k recorded, and a phage burst size calculated as $P = BS * N_i$. The new phage concentration is then given by $\phi(t + \Delta t) = (\phi(t) * v + BS * N_i)/v$ where v is the water volume under consideration.

We initialized the model with M groups of size N cells, such that each simulation has exactly the same biomass (i.e. $M * N$ is a constant across all simulations). As we know that large biomass individuals can arise from our other simulation work, we gave individuals a size that ranged from 10 cells to 10,000 cells in the group. Therefore, the number of groups in each simulation ranged from 1000 groups of 10 cells, to 1 group of $10,000$ cells. Then, we set the phage concentration, and we used the Gillespie algorithm to track the encounters that each individual had during the course of the simulation. The simulation stop condition was when all members of the population were killed, or until 120hrs had passed. When each individual was killed, they were assigned the time taken to be infected as their "livable time". Any individual that survived past 120hrs was given the time $\tau_L = 120$ hrs, aka the max simulation time. We then averaged these livable times across all the individuals. To account for the different population sizes of each simulation, some simulations were run multiple times to acquire statistical averaging power. So for group sizes: [10, 30, 100, 300, 1000, 3000, 10,000], the number of simulations run was, respectively: [1,1,1,3,10,30,100].

References

[1] Jonasz Słomka et al. “Encounter rates prime interactions between microorganisms”. In: *Interface Focus* 13.2 (Feb. 2023). Publisher: Royal Society, p. 20220059. DOI: 10.1098/rsfs.2022.0059. URL: https://royalsocietypublishing.org/doi/full/10.1098/rsfs.2022.0059 (visited on 10/10/2024).

[2] Thomas Kiørboe and E. Saiz. “Planktivorous feeding in calm and turbulent environments, with emphasis on copepods”. English. In: *Marine Ecology - Progress Series* 122.1-3 (1995). Publisher: Inter Research, pp. 135–145. ISSN: 0171-8630. DOI: 10.3354/meps122135.

[3] Michael A Delichatsios and Ronald F Probstein. “Coagulation in turbulent flow: Theory and experiment”. en. In: *Journal of Colloid and Interface Science* 51.3 (June 1975), pp. 394–405. ISSN: 00219797. DOI: 10.1016/0021-9797(75)90135-6. URL: https://linkinghub.elsevier.com/retrieve/pii/0021979775901356 (visited on 09/16/2024).

[4] Eric J. P. Woittiez, Harm J. J. Jonker, and Luís M. Portela. “On the Combined Effects of Turbulence and Gravity on Droplet Collisions in Clouds: A Numerical Study”. In: *Journal of the Atmospheric Sciences* 66.7 (2009), pp. 1926–1943. DOI: 10.1175/2005JAS2669.1. URL: https://journals.ametsoc.org/view/journals/atsc/66/7/2005jas2669.1.xml.

[5] Keivan Kaveh and Andreas Malcherek. “Settling velocity characteristics of inertial particles in turbulent and wave-induced environments”. In: *International Journal of Multiphase Flow* 179 (2024), p. 104930. ISSN: 0301-9322. DOI: https://doi.org/10.1016/j.ijmultiphaseflow.2024.104930. URL: https://www.sciencedirect.com/science/article/pii/S0301932224002076.

[6] Jonasz Słomka et al. “Encounter rates between bacteria and small sinking particles”. en. In: *New Journal of Physics* 22.4 (Apr. 2020), p. 043016. ISSN: 1367-2630. DOI: 10.1088/1367-2630/ab73c9. URL: https://iopscience.iop.org/article/10.1088/1367-2630/ab73c9 (visited on 05/09/2025).

[7] Amin Dehkharghani et al. “Bacterial scattering in microfluidic crystal flows reveals giant active Taylor–Aris dispersion”. In: *Proceedings of the National Academy of Sciences* 116.23 (2019), pp. 11119–11124. DOI: 10.1073/pnas.1819613116. eprint: https://www.pnas.org/doi/pdf/10.1073/pnas.1819613116. URL: https://www.pnas.org/doi/abs/10.1073/pnas.1819613116.

[8] P. G. Saffman and J. S. Turner. “On the collision of drops in turbulent clouds”. en. In: *Journal of Fluid Mechanics* 1.01 (May 1956), p. 16. ISSN: 0022-1120, 1469-7645. DOI: 10.1017/S0022112056000020. URL: http://www.journals.cambridge.org/abstract_S0022112056000020 (visited on 04/03/2025).

[9] Hans R. Pruppacher and James D. Klett. *Microphysics of clouds and precipitation.* D. Reidel Publishing Company, 1978.

[10] Alice L. Alldredge and Chris Gotschalk. “In situ settling behavior of marine snow”. en. In: *Limnology and Oceanography* 33.3 (May 1988), pp. 339–351. ISSN: 0024-3590, 1939-5590. DOI: 10.4319/lo.1988.33.3.0339. URL: https://aslopubs.onlinelibrary.wiley.com/doi/10.4319/lo.1988.33.3.0339 (visited on 06/03/2025).

[11] Richard W. Eppley, Robert W. Holmes, and John D.H. Strickland. “Sinking rates of marine phytoplankton measured with a fluorometer”. en. In: *Journal of Experimental Marine Biology and Ecology* 1.2 (Jan. 1967), pp. 191–208. ISSN: 00220981. DOI: 10.1016/0022-0981(67)90014-7. URL: https://linkinghub.elsevier.com/retrieve/pii/0022098167900147 (visited on 06/03/2025).

[12] Jochen Büchs et al. “Power consumption in shaking flasks on rotary shaking machines: I. Power consumption measurement in unbaffled flasks at low liquid viscosity”. en. In: *Biotechnology and Bioengineering* 68.6 (June 2000), pp. 589–593. ISSN: 0006-3592, 1097-0290. DOI: 10.1002/(SICI)1097-0290(20000620)68:6<589::AID-BIT1>3.0.CO;2-J. URL: https://onlinelibrary.wiley.com/doi/10.1002/(SICI)1097-0290(20000620)68:6%3C589::AID-BIT1%3E3.0.CO;2-J (visited on 06/03/2025).

[13] Jeannie Gardner and Gary Tatterson. “Characterization of mixing in shaker table containers”. In: *Biotechnology and Bioengineering* 39.7 (1992), pp. 794–797. DOI: https://doi.org/10.1002/bit.260390713.

[14] Paul E. Dimotakis. “The mixing transition in turbulent flows”. In: *Journal of Fluid Mechanics* 409 (2000), pp. 69–98. DOI: 10.1017/S0022112099007946.

[15] Philip J. Wiles et al. “A novel technique for measuring the rate of turbulent dissipation in the marine environment”. In: *Geophysical Research Letters* 33.L21608 (2006). DOI: 10.1029/2006GL027050.

[16] A. D. Greene, P. J. Hendricks, and M. C. Gregg. “Using an ADCP to Estimate Turbulent Kinetic Energy Dissipation Rate in Sheltered Coastal Waters”. In: *Journal of Atmospheric and Oceanic Technology* 32.2 (2015), pp. 318–333. DOI: 10.1175/JTECH-D-13-00207.1. URL: https://journals.ametsoc.org/view/journals/atot/32/2/jtech-d-13-00207_1.xml.

[17] P.R. Renosh et al. “High frequency variability of particle size distribution and its dependency on turbulence over the sea bottom during re-suspension processes”. In: *Continental Shelf Research* 77 (2014), pp. 51–60. DOI: 10.1016/j.csr.2014.01.024.

[18] Hao Xun et al. “Variability of turbulent mixing observed by high-resolution Acoustic Doppler Current Profilers in the deep South China Sea”. In: *Frontiers of Marine Science* 12 (2025). DOI: 10.3389/fmars.2025.1643170.

[19] Jacques Monod. “THE GROWTH OF BACTERIAL CULTURES”. In: *Annual Review of Microbiology* 3.Volume 3, 1949 (1949), pp. 371–394. ISSN: 1545-3251. DOI: https://doi.org/10.1146/annurev.mi.03.100149.002103. URL: https://www.annualreviews.org/content/journals/10.1146/annurev.mi.03.100149.002103.

[20] André W. Visser and Thomas Kiørboe. “Plankton motility patterns and encounter rates”. en. In: *Oecologia* 148.3 (June 2006), pp. 538–546. ISSN: 0029-8549, 1432-1939. DOI: 10.1007/s00442-006-0385-4. URL: http://link.springer.com/10.1007/s00442-006-0385-4 (visited on 03/10/2025).
